# Migration distance affects how closely Eurasian wigeons follow spring phenology during migration

**DOI:** 10.1186/s40462-021-00296-0

**Published:** 2021-12-11

**Authors:** Mariëlle L. van Toor, Sergey Kharitonov, Saulius Švažas, Mindaugas Dagys, Erik Kleyheeg, Gerard Müskens, Ulf Ottosson, Ramunas Žydelis, Jonas Waldenström

**Affiliations:** 1 Centre for Ecology and Evolution in Microbial Model Systems, Linnaeus University, Kalmar, Sweden; 2 A. N. Severtsov Institut of Ecology and Evolution RAS, Moscow, Russia; 3 Nature Research Centre, Vilnius, Lithuania; 4 Sovon Dutch Centre for Field Ornithology, Nijmegen, The Netherlands; 5 Müskens Fauna, Groesbeek, The Netherlands; 6 Ornitela UAB, Vilnius, Lithuania

**Keywords:** Arrival timing, Herbivore, Hidden Markov model, *Mareca penelope*, Migration timing, Thermal growing season, Telemetry

## Abstract

**Background:**

The timing of migration for herbivorous migratory birds is thought to coincide with spring phenology as emerging vegetation supplies them with the resources to fuel migration, and, in species with a capital breeding strategy also provides individuals with energy for use on the breeding grounds. Individuals with very long migration distances might however have to trade off between utilising optimal conditions en route and reaching the breeding grounds early, potentially leading to them overtaking spring on the way. Here, we investigate whether migration distance affects how closely individually tracked Eurasian wigeons follow spring phenology during spring migration.

**Methods:**

We captured wigeons in the Netherlands and Lithuania and tracked them throughout spring migration to identify staging sites and timing of arrival. Using temperature-derived indicators of spring phenology, we investigated how maximum longitude reached and migration distance affected how closely wigeons followed spring. We further estimated the impact of tagging on wigeon migration by comparing spring migratory timing between tracked individuals and ring recovery data sets.

**Results:**

Wigeons migrated to locations between 300 and 4000 km from the capture site, and migrated up to 1000 km in a single day. We found that wigeons migrating to more north-easterly locations followed spring phenology more closely, and increasingly so the greater distance they had covered during migration. Yet we also found that despite tags equalling only around 2% of individual’s body mass, individuals were on average 11–12 days slower than ring-marked individuals from the same general population.

**Discussion:**

Overall, our results suggest that migratory strategy can vary dependent on migration distance within species, and even within the same migratory corridor. Individual decisions thus depend not only on environmental cues, but potentially also trade-offs made during later life-history stages.

**Supplementary Information:**

The online version contains supplementary material available at 10.1186/s40462-021-00296-0.

## Background

The timing of major life history events can affect the reproductive fitness and survival of the individual [1, 2]. Birds in particular are known to time the energetically demanding breeding season to the peak of suitable foraging conditions (e.g. [3, 4]). The consequences of phenological mismatches between reproduction and food availability have been highlighted through climate change in particular. The shift of peak food availability caused by an advancement of spring phenology, happening at a faster pace than species can adapt to, can affect the reproductive success of individuals [1, 5]. This may be especially prominent in highly seasonal habitat such as the Arctic, where the availability of peak foraging conditions can be particularly short [6, 7].

Year-to-year variation in environmental conditions, and the consequent variation in the timing of peak conditions already make it difficult for individuals to optimise their timing of breeding attempts. In seasonally migrant birds, however, the additional cost of movement exacerbates the trade-off between the potential risks and rewards of earlier or later arrival at the breeding grounds. But the challenge of a timely arrival is not necessarily limited to the arrival at the breeding grounds alone. Staging periods can have positive carry-over effects into the breeding period [8]. Consequently, timing the arrival at staging sites with favourable conditions can influence both an individual’s survival and its success during the subsequent breeding season [9, 10]. Indeed, migratory herbivores such as arctic-breeding geese [11, 12] and migratory ungulates [13, 14] have been shown to follow the peak availability of vegetation growth during their spring migration. When individuals time staging periods with peak foraging conditions, the energy reserves accumulated en route can positively affect their reproductive success [15, 16].

However, some arctic-breeding birds have been shown to forego peak foraging opportunities on staging sites en route. Si et al. [17] found that Barnacle geese (*Branta bernicla*) initially utilised peak foraging conditions during stopovers, but later on migrated at a faster pace than spring phenology. As a result, they overtook peak foraging conditions en route to allow their offspring to utilise optimal conditions once hatched. Arctic summers only provide a short window for successful reproduction, indicating that the early arrival might be the result of a trade-off between utilising conditions en route for fat accumulation and those experienced on the breeding grounds [18]. This would also suggest that migratory distance should affect migration speed. Specifically, individuals that migrate over long distances and/or breed in areas with short seasons should migrate faster [19] than short-distance migratory conspecifics, which would be able to minimise the energy spent on migration by migrating slower [20]. Indeed, observations in adult waders and Northern wheatears (*Oenanthe oenanthe*) showed that individuals migrating over short distances approximate an energy-minimising, slower migration strategy, whereas long-distance migratory conspecifics reduced the time spent on migration [21, 22].

In this study, we extend on these previous observations by investigating how migratory distance affects the timing of migration in relation to spring phenology. Investigating how within-species variation in migratory distance affects en-route decision-making, especially in relation to environmental conditions, is important especially in the light of the changes to spring phenology. Longer migratory distance and shorter breeding seasons seem to come with temporal constraints, forcing individuals to migrate faster than over shorter distances [21, 22]. Thus, long-distance migrants might be more susceptible to the advancement of spring and the increased year-to-year variation of spring conditions brought on by climate change. Recent examples have shown that long-distance migratory species might be more strongly affected by climate change [5, 23], and experience population declines [24]. Studying how migratory distance affects the timing of migration within a single species might thus provide some insight into how changes in environmental conditions might affect individuals of the same species differently depending on the challenges faced during migration and on the breeding grounds.

Here, we address this question in an avian herbivore, the Eurasian wigeon (*Mareca penelope*, hereafter wigeon). The wigeon is a medium sized dabbling duck and uniquely suited for investigating within-species variation of spring migratory timing: wigeons are predominantly herbivorous and migrate early in spring [25–27]. The availability of suitable conditions along their migration should thus be tightly linked to plant phenology, and similar to geese it can be expected that wigeons follow phenology during spring migration. In contrast to geese, however, wigeons are thought to be income rather than capital breeders [28]. As income breeders, the arrival of wigeons at the breeding grounds should be directly coupled to suitable conditions as they rely on exogenous resources for their sustenance [29, 30]. Wigeons furthermore have a very broad distribution range [25, 26]. They display a large variation in spring migratory distances, with birds wintering in the Netherlands spending the summer months in an area ranging from western Denmark to the Russian Arctic (see https://www.vogeltrekatlas.nl [31]).

We captured and tagged wigeons in two locations, and followed their spring migratory movements during 3 years. Identifying staging sites used by individuals throughout spring migration, we determined the date of arrival for both wigeons and an indicator for spring phenology at each staging site. Here, we used the onset of the thermal growing season [32] ($TGS_{\mathrm{onset}}$) as an indicator for spring phenology. The thermal growing season (TGS) signifies the period of the year when the mean daily temperature lies above a defined threshold temperature, chosen to reflect the temperature above which growth of relevant vegetation occurs. The day that marks the onset of the thermal growing season $TGS_{\mathrm{onset}}$ hence is the day when daily mean temperature rises above the threshold temperature. This metric differs from other indicators of spring phenology used in similar studies as it marks the beginning of vegetation growth, rather than when peak growth or forage availability occurs (e.g. green wave model [13] and jerk of growing degree days [12]). We expected that tracked wigeons might be slowed down by the additional weight and drag of tracking devices [33–36], so that their arrival might not reflect optimal or near-optimal conditions at staging sites. We thus decided to study the effect of migratory distance on how long after the $TGS_{\mathrm{onset}}$ wigeons would arrive at staging sites rather than characterising the environmental conditions upon arrival. Here, we used the number of absolute days between $TGS_{\mathrm{onset}}$ and wigeon arrival ($\Delta {\text{arrival}}_{\mathrm{d}}$) to estimate whether migration speed relative to spring phenology changes with migratory distance (see Additional files 1, 2 and 3 for an alternative approach using growing degree days [12, 37]). We additionally compared the progress of spring migration of tracked wigeons with long-term ring recovery data to estimate the effect of tagging on wigeon migration. Overall, this study provides valuable insight into how individuals might have to trade-off between saving energy and saving time during migration depending on the distance between the wintering and breeding grounds. Following previous findings [17, 21, 22], we expected that wigeons migrating to the Arctic should follow spring more closely than their short-distance migratory conspecifics.

## Methods

### Capture and tagging of Eurasian wigeons

Eurasian wigeons were captured with cannon nets in two locations: a polder near Krommeniedijk in the Netherlands (NLD, wintering site), and a seasonally flooded meadow in the Nemunas Delta Regional Park in Lithuania (LTU, staging site). Fieldwork was performed during February 2018 and March 2019 (NLD), and during April 2018 and March 2019 (LTU). After capture, individuals were kept in a soft-cloth tent until processed. All individuals were weighed, measured, as well as marked with steel rings with unique identifying numbers. Birds were sexed and aged using visual assessment. Dutch individuals additionally had fecal samples taken to test for infection with avian influenza viruses and *Campylobacter* sp., and were marked with colour rings as part of a colour ring read-out study.

We selected apparently healthy individuals with a body mass > 600 g for deployment with Ornitela OrniTrack-15 GPS/GSM tags (tag weight: 15 g). In total, we tagged 39 individuals (14 females, 25 males; 25 in NLD, 14 in LTU) with body mass ranging from 650 to 880 g (Table 1, Additional file 1: Table S1). The tags were glued onto a foam pad, and attached to wigeons with individually fitted backpack harnesses made from 6.5 mm tubular teflon tape. The weight of the tag itself was equivalent to a mean of 2.03% of the body mass of individuals, and with the addition of the custom harnesses ($\sim$ 3 g) the total weight deployed never exceeded 2.8% of the body mass of individuals. After tagging, individuals were released within 200 m of the capture site.

**Table 1:** Sample sizes for the individuals that were captured and tagged in Lithuania and the Netherlands, broken down by year of capture and sex of individuals

	Year	Deployment date	n individuals	n females	n males
Netherlands	2018	Feb 05	20	6	14
	2019	Mar 16	5	5	0
Lithuania	2018	Apr 26	9	3	6
	2019	Mar 29 – 31	5	0	5
Total tagged			39	14	25
Available for analysis			27	12	15

Also included is the sample size with respect to individuals that were available for the modelling of arrival timing. More details on tracking duration and number of locations per individual and year are available in Additional file 1: Table S1

### Sampling schedule and data pre-processing

Tags were programmed to record 15s bursts of 1 Hz GPS-locations once every hour at battery levels > 75%. Sampling schedules for battery levels < 75% were multiples of the highest possible sampling frequency of $1\,{\text{h}}^{-1}$ and could thus be counted as missed fixes (Additional file 1: Table S2).

The tracking data were transferred via GSM (when available) every 12 h, and forwarded automatically to the animal movement database Movebank (https://www.movebank.org). We downloaded the data on 2020-06-01 (available under DOI 10.5441/001/1.dv5mm289 [38], see also Additional file 1: Fig. S1). We cleaned the data by removing inactive locations (ground speed = 0 m/s) from the end of each track, and removed the bursts of GPS locations prior to analyses. We inserted missed fixes (with unknown location) to near-regularise trajectories, and calculated solar time as fractional hours relative to solar midnight at each observation. We kept the initial highest sampling frequency of 1 location per hour as initial inspection of the data revealed that wigeons might interrupt their migratory movements for durations < 24 h. Finally, we split individuals by year of observation. Details of the data pre-processing are available in the Additional file 2.

### Identifying migratory movement and spring staging sites

We determined the spring migratory period for each individual and year by identifying the start and end location of their migratory movements. We here term the locations start and end rather than wintering and breeding as the individuals captured in the Nemunas delta were likely not wintering there. Breeding locations could also not be determined with certainty, and some individuals were shot or predated prior to reaching their breeding areas. We defined the starting location as the south-westernmost location used by an individual during February through April, and the end location as the north-easternmost location reached during May through July. The start of spring migration was determined as the time when individuals moved away from the starting location above a certain threshold distance $d_{t}$ without returning, and the end of migration as the time when individuals were first observed within $d_{t}$ of the respective end location. We defined $d_{t}$ using the airspeed estimates of migratory wigeons provided in [39], with $d_{t} = (\overline{v_{a}} - 2\sigma ) \times 3600 = 50.184$ km, with $\overline{v_{a}}$ being the mean reported airspeed (in m/s), $\sigma$ being the standard deviation, so $d_{t}$ equals the distance a migratory wigeon at a speed of mean − 2 $\times$ s.d. would be expected to fly within 1 h in the absence of wind.

To assist in identifying staging periods in the spring migratory tracks, we used a hidden Markov model (HMM) to estimate the behavioural states and corresponding movement parameters from the tracking data. We implemented HMMs using the momentuHMM-package for R (package version 1.5.3) developed by McClintock and Michelot [40]. This implementation was specifically developed for regular animal movement trajectories, and allows for the estimation of behavioural states from user-specified movement parameters. The final model used square-root transformed step lengths in km (gamma distribution) and turning angles in radians (Weibull distribution) to estimate the probabilities of locations being in one of four movement states, specifically *rest* (state 1) indicating inactivity, *non-flight* (state 2) whenever individuals were active on the ground (e.g. foraging), *local flight* (state 3) indicated by lower step lengths and lower directional persistence than *migratory flight* (state 4). We further included solar time of day as a cyclical covariate to express probabilities of state switching as a function of time of day. This model was deemed best for the purpose of this study after visual assessment of the fitted distributions for all movement parameters and states.

We classified each location with the most likely behavioural state using the Viterbi algorithm [41], and excluded any locations considered migratory from the data. Finally, we then calculated the geodesic distance between subsequent locations of the remaining non-migratory observations. Whenever this distance exceeded $d_{t}$, we considered the corresponding locations as a new staging site, and assigned a unique identifier to each staging site. We considered the first day of observations at a staging site as an arrival event. We were not able to ascertain breeding locations for most individuals, and could thus not with certainty determine the end of spring migration. Wigeons are thought to initiate breeding up until mid-June. Given the potential delay of tracked wigeons due to the transmitter, we assumed that tracked wigeons would arrive latest June 30. We excluded all arrival events that were within $d_{t}$ from the starting location, and every arrival event that occurred after June 30 of the respective year.

Details about model fitting, the final 4-state HMM, and the segmentation of non-migratory locations can be found in Additional file 1 (Additional file 1: Figs. S2 and S3) and Additional file 2.

### Environmental data

We collected environmental data to derive potential predictors of wigeon arrival time. Specifically, we acquired hourly ECMWF ERA5 climate re-analysis data on temperature for the years 1998–2019 from the Copernicus Climate Data Store [42]. We initially calculated daily mean temperature using temperature at 2 m above ground, and determined the start of the thermal growing season with a reference temperature of 5 $^{\circ }{\mathrm{C}}$ according to Ruosteenoja et al. [32]. We chose 5 $^{\circ }{\mathrm{C}}$ as it is a commonly used reference temperature for cool-season pasture and crops [43]. Using only the onset for the thermal growing season ($TGS_{\mathrm{onset}}$) for the years 1998–2017, we calculated the mean $TGS_{\mathrm{onset}}$ for this period; hereafter $TGS_{\mathrm{mean}}$. We then subtracted the $TGS_{\mathrm{mean}}$ from the $TGS_{\mathrm{onset}}$ of each study year (2018, 2019, 2020) to determine how the given year deviated from the long term average, hereafter $TGS_{\mathrm{deviation}}$. Positive values of $TGS_{\mathrm{deviation}}$ indicate that the $TGS_{\mathrm{onset}}$ arrived later than average, and vice versa.

In addition to the $TGS_{\mathrm{onset}}$, we also derived growing degree days for all study years. We again used a reference temperature of 5 $^{\circ }{\mathrm{C}}$, by subtracting the reference temperature from the mean daily temperature. Negative growing degrees were set to zero. All subsequent analyses were repeated using the growing degree days accumulated between the $TGS_{\mathrm{onset}}$ and an arrival event. As the implications from these analyses were equivalent to the analyses using $TGS_{\mathrm{onset}}$, the methodology and results for these alternatives are presented in the Appendix only (Additional files 1 and 2).

Code for the processing of all environmental data products can be found in Additional file 3.

### Annotation of arrival events and arrival delay

We annotated all arrival events with the corresponding $TGS_{\mathrm{onset}}$ and $TGS_{\mathrm{deviation}}$ for the respective year. We further re-projected all wigeon arrival locations using a custom two-point equidistant projection (Additional file 2; hereafter longitude and latitude). We determined for each individual the highest longitude it reached during that spring migration (in two-point equidistant projection; here after “maximum longitude”), as an indication. Longitude and latitude at arrival sites were highly correlated due to the north-easterly direction of migration, and we chose longitude as the range of longitudes observed in arrival locations was greater than the range of latitudes. Finally, we calculated the cumulative geodesic distance between subsequent arrival events, starting at the respective starting location (hereafter “distance traveled”). We scaled and centered all of the above variables.

We then determined arrival delay relative to spring phenology $\Delta \text {arrival}_{\mathrm{d}}$, which we defined as the difference between the $TGS_{\mathrm{onset}}$ and an arrival event. We excluded all arrival events for which environmental information were not available, leaving a data set containing 208 arrival events for $\Delta \text {arrival}_{\mathrm{d}}$. The data sets contained arrival events for 28 wigeons, with three individuals having been observed during two spring migratory seasons.

### Models for wigeon arrival timing

We used linear mixed-effects regression to model wigeon arrival delay relative to the onset of the thermal growing season. During the visual exploration of the data set, we found that several of the potential predictor terms were highly correlated, such as migration distance, longitude, and day of the year (range of Pearson correlation coefficient $r{:} -\,0.48 - \,0.85$). Consequently, including all these terms in a single model would limit the conclusions we could draw from the model. We thus compared these terms in isolation, and decided to include (1) $TGS_{\mathrm{deviation}}$ to account for differences between study years, (2) maximum longitude reached by individuals as an indicator of migratory distance, and (3) a three-way interaction term between maximum longitude, distance traveled, and capture site in the models to estimate how the effect of total migratory distance expressed as maximum longitude changes with distance traveled and capture site, and (4) capture site to estimate potential differences between individuals captured in the Netherlands and Lithuania. Since the data set contained repeated observations of individuals, and thus unobserved heterogeneity between individuals relating to arrival timing, we included year of observation nested in the individual identifier as a random effect. Recognising that our longitudinal data set further included interdependence of repeated observations, we extended the conditional model with an Ornstein–Uhlenbeck correlation structure, with arrival day as term and individual-year as grouping factor.

We used maximum longitude as an indicator of total migratory distance as we did not know the wintering location of individuals captured in Lithuania, although ring recoveries and indeed the data collected for this study suggest that birds from both capture sites share the same general wintering areas (see Additional file 1: Fig. S1 and [38]). Yet we cannot be certain of their wintering locations during the first year of observation. We also decided to use maximum longitude rather than maximum latitude as the arrivals varied to a greater extent in longitude than latitude leading to a higher coefficient of determination for the model (see Additional file 2 for an alternative model with maximum latitude).

Using exploratory models containing only sex or capture site as fixed effect terms, we found that neither contributed much to explaining arrival delay (see Additional file 2). We thus excluded capture site and sex from the final models. The final model contained, apart from the conditional model, $TGS_{\mathrm{deviation}}$, maximum longitude reached by individuals, and an interactive term between maximum longitude, capture site, and distance traveled as independent predictors.

Despite accounting for auto-correlation of repeated observations of individuals within the same year, we wanted to ensure our results were not an artifact of residual auto-correlation, or other interdependence of the observations. We did so by supplementing above models with additional models fitted on only the last arrival event for each individual and year. Since retaining only a single event for each individual and year, the data were no longer longitudinal in nature. We thus removed the correlation structure from the model, and removed the interaction for maximum longitude and distance traveled.

All models were implemented in R using the package glmmTMB [44] (see Additional file 2 for details).

### Estimating effects of tagging on migratory timing

Finally, we investigated the potential effects of the GPS/GSM transmitters on the timing of spring migration by comparing the tagged individuals with wigeon ring recovery data. For this purpose, ring recovery data from wigeons either marked or recovered within the scope of the ringing schemes Arnhem (Netherlands, n = 472), London (Great Britain, n = 452), and Moscow (Russia, n = 140) were available to us. We used these data to determine the mean date of passage of wigeons through longitudinal bands along the migratory flyway. We defined longitudinal bands with a width of 10$^\circ$ longitude each, spanning from 0$^\circ$ E to 80$^\circ$ E, covering the entire span of longitudes observed for the tracking data ($\sim$ 4.5–80$^\circ$ longitude). While the ring recovery data spanned a wider range of longitudes, longitudinal bands outside the range of tracked wigeons were excluded. We computed the mean and s.d. for recovery dates of ring recoveries during spring (from/to) per band (See Additional file 1: Fig. S4). For ring recoveries from Russia, we additionally used ten control points, (wigeons marked during the spring migratory period, rather than just recoveries).

We then determined, for each individual and year, the date of the first arrival event surpassing the lower longitude for each band, if at all. We then calculated the difference between dates for the individual and the mean date observed in the ringing data for each band and ringing scheme (hereafter $\Delta {\text{arrival}}_{ring}$). Finally, we investigated whether delay relative to the ringing data could be explained by sex and/or capture site using linear mixed-effects regression, further including longitude and the corresponding $TGS_{\mathrm{deviation}}$ as a model term to account for whether the TGS arrived earlier or later than average during the previous 20 years.

The model was implemented in R using the package glmmTMB [44], and details are available in Additional file 4.

## Results

### Wigeon spring migratory trajectories

**Fig. 1:**
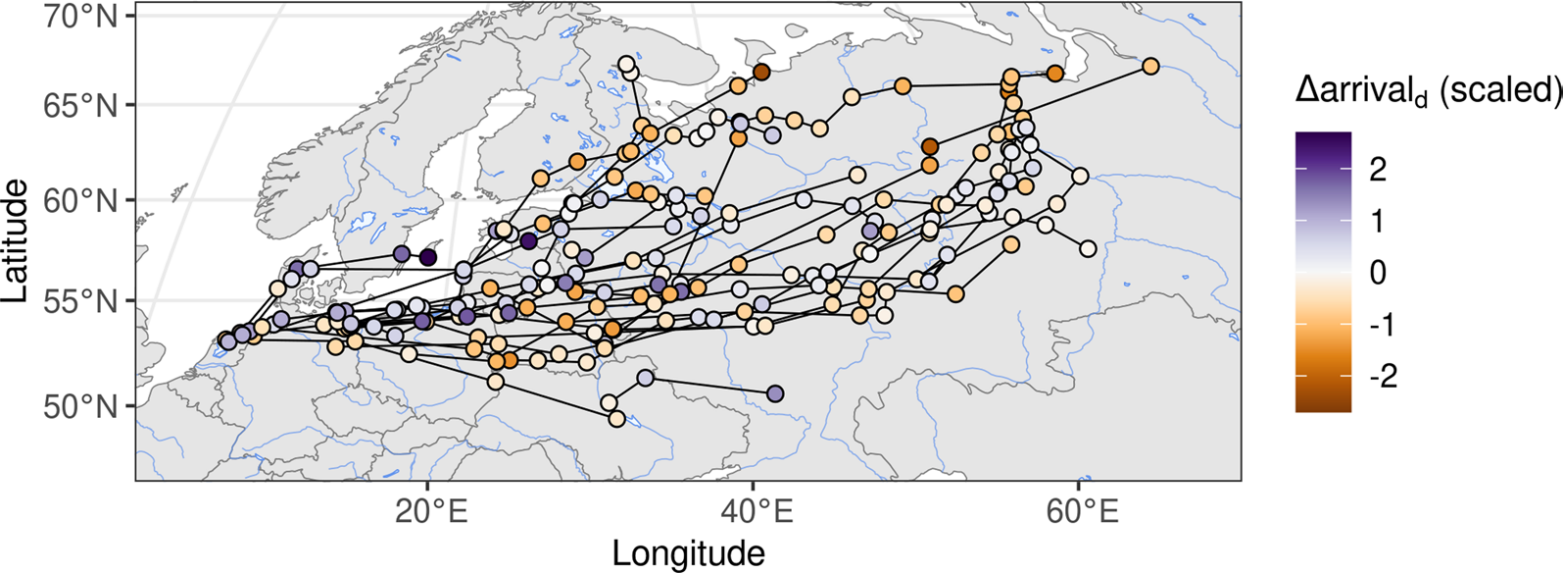
Arrival events at staging sites relative to the ${\text{TGS}}_{\mathrm{onset}}$ Each arrival event is shown as point coloured according to the (scaled) $\Delta {\text{arrival}}_{\mathrm{d}}$, with arrival events early relative to the $TGS_{\mathrm{onset}}$ shown in orange, and arrivals occurring later relative to the $TGS_{\mathrm{onset}}$ shown in purple. Subsequent arrival events recorded for the same individual and year are connected by lines. Data are shown in interrupted Goode Homolosine projection

**Fig. 2:**
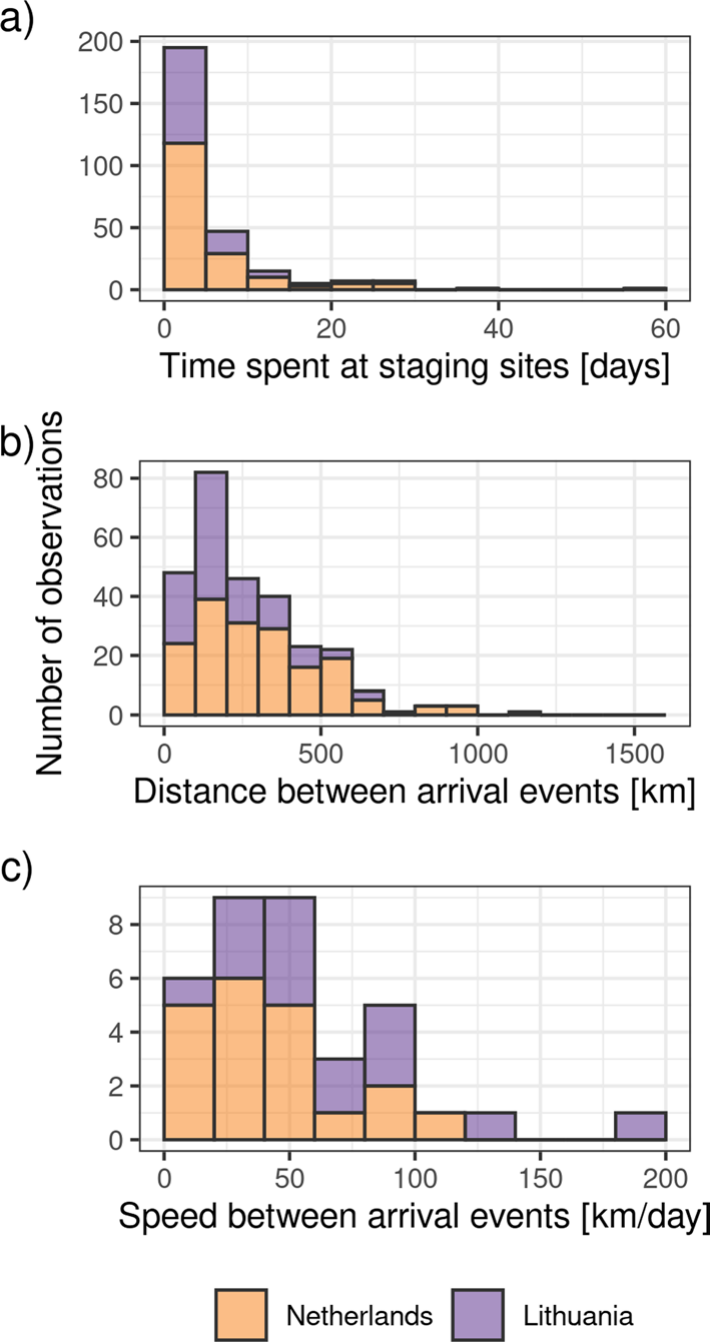
Summaries for staging duration, and distance and speed between subsequent staging sites. **a** shows the time that individuals spent at staging sites in days; **b** shows geodesic distance between subsequent staging sites in kilometers; and **c** shows how fast individuals traveled between staging sites, calculated as the distance shown in (**b**) divided by the time between the last location in one staging site, and the first location recorded in the subsequent staging site

After data preparation, 35 spring migratory trajectories from 31 individuals remained, with the decrease in individuals being due to loss of tags and predation. Wigeons migrated along a broad front with an initially eastward direction. Long-distance migratory individuals turned to a more northerly direction after passing the southern end of the Ural Mountains (Fig. 1 and Additional file 1: Fig. S1). The data suggest that the southwestern-most area used during the summer was in SW Denmark (male "5512398"), and the northeastern-most end locations were located in the Ob River Delta and on the river Taz (several individuals). The longest geodesic distance between any starting and end location, after filtering, was 4184 km (median: 1899 km; Q1: 1155 km; Q3: 3130 km). During spring migration, we found that wigeons covered up to 963 km per day, and moved with median speed of 48.2 km/day (Q1 = 30.0; Q3 = 60.9 km/day) during migration (Fig. 2).

### Environmental conditions at time of arrival

We found that individuals arrived at staging sites with a median of 22.5 days (Q1 = 13; Q3 = 35.3 days) after the onset of the thermal growing season. The mixed effects regression models revealed that $\Delta {\text{arrival}}_{\mathrm{d}}$ varied with maximum longitude, with individuals of both capture sites reaching higher longitudes (i.e., more north-easterly locations) arriving relatively earlier compared to $TGS_{\mathrm{onset}}$. This effect increased in size the further individuals had already traveled along their migration irrespective of capture site (interaction term). We further found that $TGS_{\mathrm{deviation}}$ had a negative effect on $\Delta {\text{arrival}}_{\mathrm{d}}$ (see Table 2). Overall, we found that the inclusion of $TGS_{\mathrm{deviation}}$, maximum longitude, and distance traveled could explain about 42% of the variation observed in $\Delta \text {arrival}_{\mathrm{d}}$ (marginal $R^{2}\,=\,0.42$ [45]). As scaled predictors and response terms do not allow for direct evaluation of the biological magnitude of the estimates, we predicted $\Delta {\text{arrival}}_{\mathrm{d}}$ for four hypothetical breeding locations, and unscaled the result. Specifically, we made population-level predictions for hypothetical individuals wintering in the Netherlands, and set $TGS_{\mathrm{deviation}}$ to 0 and distance traveled to 1000 km. According to the prediction, $\Delta {\text{arrival}}_{\mathrm{d}}$ would be 39 days for individuals breeding in the Lithuanian capture site, 33 days for breeding around Moscow, 26 days for a breeding location near Perm, and 22 days for individuals breeding in the Ob River Delta.

**Table 2:** Below we detail the estimates with 95% confidence intervals and model statistic for the fixed effects included in the model for wigeon arrival timing relative to the $TGS_{\mathrm{onset}}$ using $\Delta {\text{arrival}}_{\mathrm{d}}$

	Estimate	95% CI	z-value
Intercept (Lithuania)	0.06	− 0.37 to 0.49	0.27
Intercept (Netherlands)	0.52	0.17 to 0.87	2.91
Deviation from $TGS_{\mathrm{onset}}$	− 0.30	− 0.35 to − 0.25	− 12.20
Maximum longitude	− 0.48	− 0.71 to − 0.24	− 3.96
Max. longitude: distance traveled (Lithuania)	− 0.17	− 0.33 to − 0.01	− 2.08
Max. longitude: distance traveled (Netherlands)	− 0.26	− 0.34 to − 0.19	− 6.76

The fixed effects contributed to a marginal $R^2=0.42$. The response and independent model terms were scaled and centered, and the model was fitted using 208 arrival events for 28 wigeons and 3 years. More details on the conditional model, including the random effect terms and the correlation structure can be found in Additional file 1: Table S2

The models fitted on the data set reduced to only include the last arrival for each individual and year confirmed what we found for the models on the full data set, with longitude having a negative effect on $\Delta {\text{arrival}}_{\mathrm{d}}$ (see Table 3). The amount of variation explained by $TGS_{\mathrm{deviation}}$ and longitude was similar to that observed for the full models (marginal $R^2=0.44$ for $\Delta {\text{arrival}}_{\mathrm{d}}$)

Results for alternative models, and all models using growing degree days accumulated after $TGS_{\mathrm{onset}}$ as response term are reported in Additional file 1 (Additional file 1: Fig. S5, Tables S3 and S4).

**Table 3:** Below we detail the estimates with 95% confidence intervals and model statistic for the fixed effects included in the model for wigeon arrival timing relative to the $TGS_{\mathrm{onset}}$ using $\Delta {\text{arrival}}_{\mathrm{d}}$ using only the last arrivals for each individual and year

	Estimate	95% CI	z-value
Intercept	0.45	0.10–0.79	2.55
Deviation from $TGS_{\mathrm{onset}}$	− 0.26	− 0.56 to 0.04	− 1.72
Maximum longitude	− 0.59	− 0.89 to − 0.30	− 3.91

The fixed effects contributed to a marginal $R^2=0.44$. The response and independent model terms were scaled and centered, and the model was fitted using 32 arrival events for 28 wigeons and 3 years

### Effects of tagging on wigeon migration speed

**Fig. 3:**
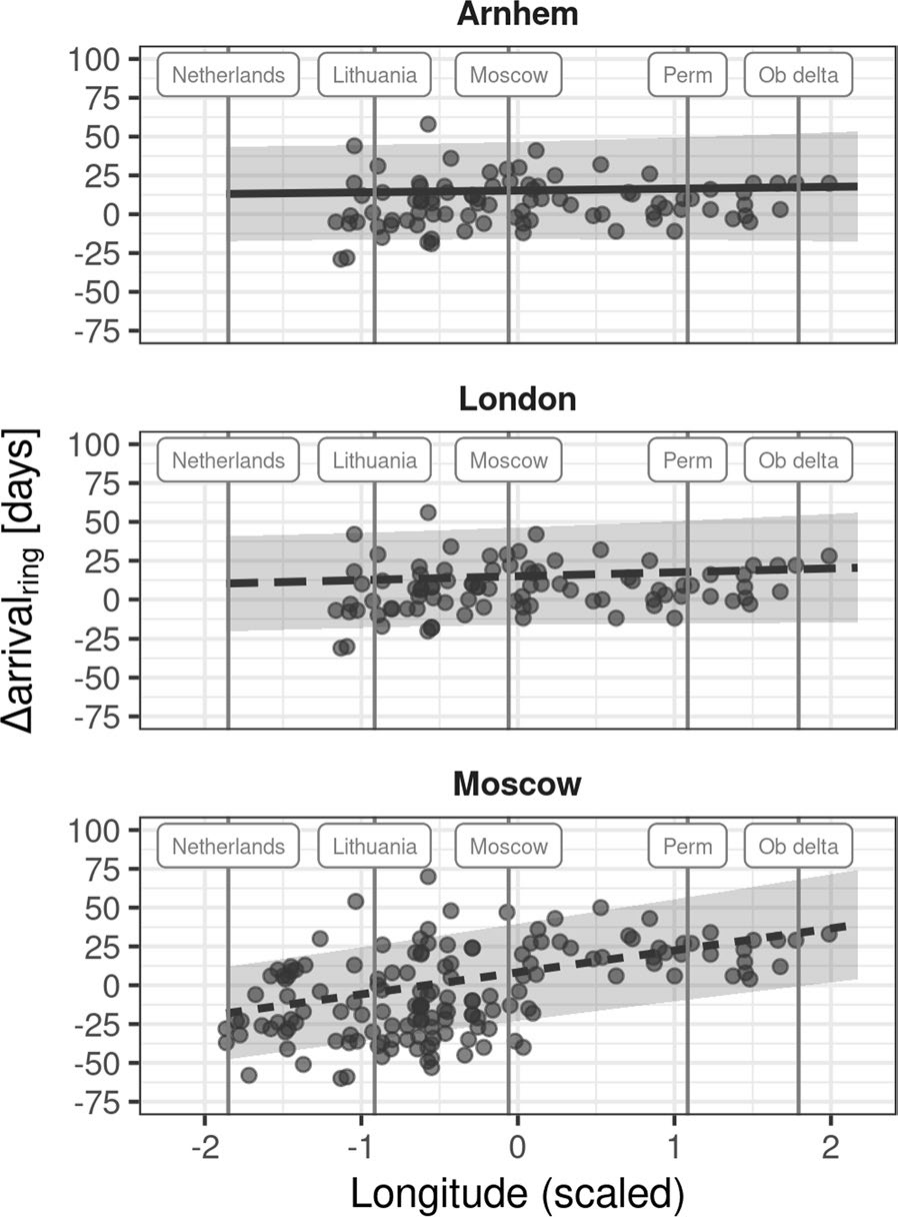
Timing of arrival compared to ring recovery data. The scatterplots show how the delay of tagged wigeons relative to ring recovery data changes of longitude. Points represent the measured $\Delta {\text{arrival}}_{ring}$, the lines and shaded areas show the estimates and 95% CIs for the effect of longitude for the respective ringing scheme. Three three panels show the data and model estimates for the ringing schemes of **a** Arnhem, **b** London, and **c** Moscow, respectively. Vertical lines and labels provide reference longitudes for locations along the migration corridor of tracked wigeons

We found that the tagged wigeons differed in migratory timing from the population of ring recoveries. Specifically, we found that the tagged wigeons lagged behind birds in the Arnhem and London ringing scheme by about 11–12 days (Arnhem: estimate = 11.57 days; 95% CI = [5.75, 17.38]; London: estimate = 11.30 days; 95% CI = [5.48, 17.12]), but only by about 5 days compared to birds in the Moscow ringing scheme (estimate = 4.64 days; 95% CI = [− 0.93, 10.21]; see Table 4). However, the model suggested that $\Delta {\text{arrival}}_{\mathrm{ring}}$ compared to Moscow ring recoveries increased with longitude, whereas $\Delta {\text{arrival}}_{\mathrm{ring}}$ compared to Arnhem and London birds remained relatively consistent across all longitudes (Fig. 3).

**Table 4:** Here we summarise the results for the model for the delay of tagged wigeons relative to ring-marked wigeons

	Estimate	95% CI	z-value
Intercept (Arnhem)	11.57	5.75 to 17.38	3.90
London	11.30	5.48 to 17.12	3.81
Moscow	4.64	− 0.93 to 10.21	1.63
Arnhem : Longitude	1.19	− 3.76 to 6.13	0.47
London : Longitude	2.51	− 2.43 to 7.45	1.00
Moscow : Longitude	14.16	10.02 to 18.29	6.70

Listed above are the estimates of $\Delta {\text{arrival}}_{\mathrm{ring}}$ for each ringing scheme and the corresponding interaction with longitude, along with 95% confidence intervals and model statistic

The model further suggested that neither sex, capture site, or deviation from the $TGS_{\mathrm{onset}}$ at arrival could substantially contribute to explaining the delay of tagged individuals compared to ringing recoveries, and were thus dropped from the model (see Additional file 4 for details).

## Discussion

**Fig. 4:**
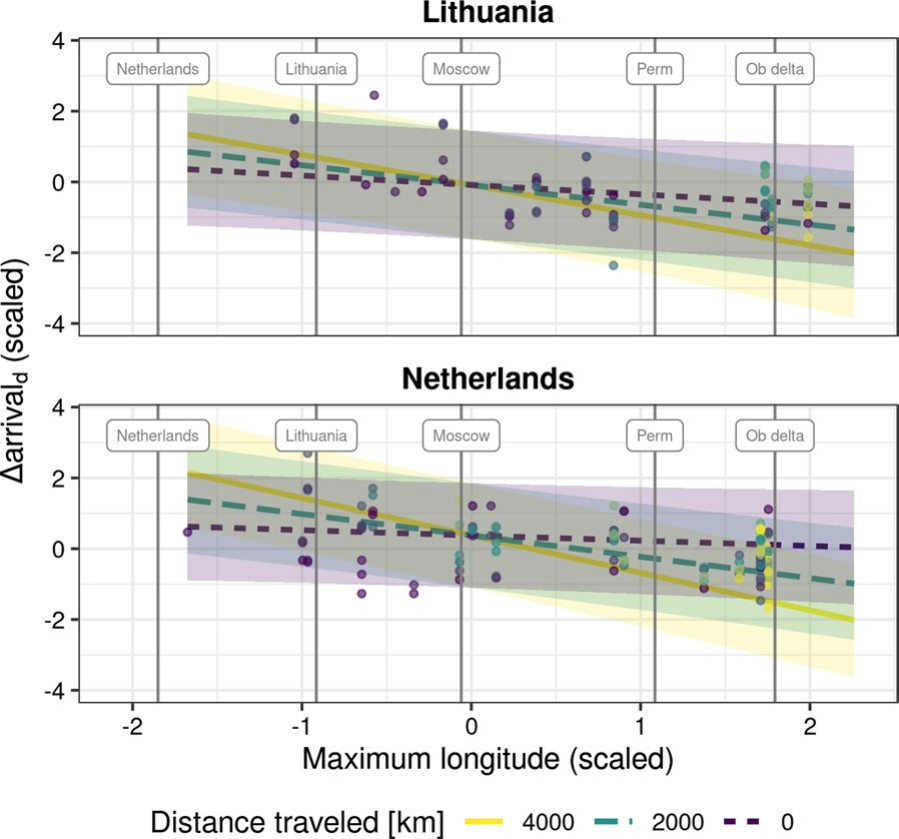
Wigeon arrival timing relative to the $\mathbf{TGS }_{\mathrm{onset}}$ Here we show the interaction effect between maximum longitude and distance traveled on $\Delta {\text{arrival}}_{\mathrm{d}}$, specifically the effect of maximum longitude for three different values of distance traveled. The lines show the estimate for the effect at the given distance, and the shaded areas reflect the 95% confidence intervals for the estimate. The data used to fit the model are shown as scatterplot. Vertical lines and labels provide reference longitudes for locations along the migration corridor of tracked wigeons

Long-distance migrants are thought to face greater temporal constraints during spring migration than species migrating over shorter distances, potentially necessitating long-distance migrants to minimise the time spent on migration, rather than energy, to reach their breeding grounds [21, 22]. In herbivorous migrants, individuals would have to follow spring phenology more closely, or indeed overtake spring [17], the longer the distance between their wintering and breeding grounds. Here, we investigated whether the spring migratory behaviour of wigeons follows this expectation. We found that individuals captured in Lithuania and the Netherlands in winter and early spring ranged from short-distance migrants (about 300 km between wintering and summering areas) to truly long-distance migrants, covering the nearly 4000 km between the Netherlands and the Ob river delta in arctic Russia. Migration distance along the overall migratory corridor of wigeons wintering in NW Europe, as estimated by maximum longitude reached, indeed affected the migratory behaviour of wigeons. Individuals breeding at higher longitudes, and given the north-east direction of migratory movements, higher latitudes, followed spring phenology more closely than their short-distance migratory conspecifics, and increasingly so the further individuals had migrated. While this effect was negligible early in migration, it became increasingly prominent over longer distances traveled (Fig. 4). Birds breeding closer to the Arctic thus arrived earlier at staging sites relative to spring phenology. This finding was consistent across capture sites despite Lithuania being a staging rather a wintering site for this species, helped by the use of maximum longitude as an indicator of migratory distance. We also found that Lithuanian individuals that were tracked into the following winter shared the general wintering area with the individuals captured in the Netherlands [38, 46].

Our findings indicate that wigeons breeding at greater distance from the wintering ground might thus experience phenologically different conditions than individuals at more southerly staging sites. This difference in $\Delta {\text{arrival}}_{\mathrm{d}}$ between birds reaching higher latitudes and longitudes as opposed to individuals supposedly breeding more southwesterly indicates that temporal constraints with regard to migration and/or breeding might increase with migratory distance. Indeed, we found that the shortest duration of the TGS for any staging site used by tracked wigeons was 110 days, as opposed to e.g. > 270 days for a potential breeding site in SW Denmark. The incubation of the eggs is thought to last about 24–25 days from laying to hatching, and about 45 days from hatching to fledging [25, 47] under good conditions. Even considering that wigeons tend to arrive after the $TGS_{\mathrm{onset}}$, a breeding attempt is decidedly shorter than the shortest growing season length observed. Compared to other species of dabbling ducks breeding in the Palaearctic, however, after arriving at the breeding site wigeons forage in flocks for up to 2–3 weeks prior to laying [48], likely to meet the energetic demands of breeding. During this period, they are very mobile and frequently move between the first ice-free plots of flooded meadows and herbaceous wetlands [49]. Wigeons might further extend their residence on the breeding grounds by replacing lost clutches [25], annual moult [50] and the subsequent need to accumulate fuel for autumnn migration. Consequently, the residence of wigeons on the breedings grounds extends beyond the breeding season alone. This does not necessarily indicate constraints imposed on individuals by the duration of the TGS, and by arriving earlier than more southerly breeding conspecifics, relative to the onset of the TGS, arctic-breeding wigeons might be able to time their breeding activity more closely with peak foraging availability.

When comparing the migratory timing of tracked wigeons to ring recovery data from three ringing schemes, we found that the wigeons tracked for this study were consistently slower than the average ring-marked population. This finding was not unexpected, as the additional weight and drag is thought to slow down migration overall [34]. We found that on average, tracked wigeons were about 11–12 days slower than individuals ringed in the Arnhem and London ringing schemes, similar to the 9.9 days of delay estimated for Northern Pintails [33]. $\Delta {\text{arrival}}_{\mathrm{ring}}$ was consistent across longitudes when compared to the Arnhem and London ringing schemes. This is slightly unexpected as the slowing effect of the tags could well accumulate due to longer staging times. We did however find that the distances covered during migratory legs became increasingly shorter over time (see Additional file 1: Fig. S6), potentially minimising the need for prolonged staging. We found that the delay relative to birds from the Moscow scheme, however, increased with longitude. While we cannot ascertain the reason for this marked difference between the London and Arnhem schemes and the Moscow scheme, we think that two potential explanations could be (1) that birds migrating towards breeding grounds in Russia might in part originate from different wintering areas, as might be indicated the overall higher longitudes reached by individuals in this ringing scheme as opposed to wigeons ringed in the Arnhem and London schemes, and (2) the great variation in mean arrival dates of Moscow birds in particular at lower longitudes, potentially due to the overall lower sample size for the Moscow ringing scheme compared to the London and Arnhem schemes (see Additional file 1: Fig. S4). During active migration, wigeons covered up to about 1000 km in non-stop flight, and maintained a mean ground speed of 20.3 m/s (median: 19.7 m/s; Q1: 16.1 m/s; Q3: 23.9 m/s). This is slightly higher than the mean reported air speed for the species [39], and suggests that tracked wigeons can maintain the same speed as un-marked individuals over long distances. Since $\Delta {\text{arrival}}_{\mathrm{ring}}$ was estimated to be consistent across longitudes, the relative changes of wigeon arrival timing relative to spring phenology should be informative for the general population, and in particular for individuals wintering in western Europe.

## Conclusions

In general, our study highlights that intra-species differences in migratory distance can affect migratory decision-making even within the same migratory corridor. Identifying the cues and constraints guiding the migratory decision-making of wild birds is pivotal to the understanding of how different and changing conditions affect not just the individual, but populations as a whole. Climate change is thought to affect long-distance migrantory species more strongly than short-distance migrants and residents [24, 51], and might lead to phenological mismatches along spring migration in, among others, arctic-breeding herbivores [34, 52]. Wigeons seem to consistently time their arrival at key sites throughout their life history with environmental cues that can be estimated from widely available data. This makes them a candidate species for estimating the potential effects of changing environmental conditions on long-distance migration [53, 54]. The species is currently considered to be undergoing a decline in population size, and the large variation in migration distance offers the opportunity to study the phenomenon within a single species, especially if smaller tags can reduce weight and drag sufficiently to reduce the tagging effects observed in this study. Climate change not only has negative consequences through increasing phenological mismatches between life history events and phenology [55, 56] but can also shift the distribution of entire populations and thus their migratory behaviour. It is for example likely that the recent shift of the main wintering grounds of wigeons breedin in Northeast European and Western Siberia from Southeast Europe to Northwest Europe was mainly caused by climate change [26]. As a consequence of the lag compared to the overall population, our study can provide limited insight into the absolute timing of wigeon spring migration in relation to spring phenology. The finding that wigeons follow spring phenology more closely further into migration should however be robust, as untagged, and thus faster wigeons, should follow the onset of the thermal growing season even more closely. In addition, this study indicates that wigeons utilise an extensive network of staging sites, which should be taken into consideration for the conservation and appropriate management of the species, as has recently been shown for wigeons migrating via the East Asian flyway [57]. Further tracking studies on wigeons in Eurasia could enable the designation and sustainable management of their key stop-over sites in the East Atlantic flyway, and beyond.

## Supplementary information

**Additional file 1.** Additional figures and tables.

**Additional file 2.** Analysis of wigeon arrival timing (R-code)

**Additional file 3.** Environmental data preparation (R-code).

**Additional file 4.** Estimating effect of tagging on arrival timing (R-code).

## Data Availability

The tracking data collected for this study are available on the Movebank Data Repository under DOI 10.5441/001/1.dv5mm289.
