# Supplementary material for: Migration distance affects how closely Eurasian wigeons follow spring phenology during migration
Source: Mov Ecol. 2021 Dec 11;9:61. doi: 10.1186/s40462-021-00296-0 (PMC8665524; doi:10.1186/s40462-021-00296-0)
Supplement: Supplementary file 1 — Additional file 1. Additional figures and tables. [file 40462_2021_296_MOESM1_ESM.pdf]

Additional File 1: Additional figures and tables

Supplementary Tables

Table S 1: Details for tagged individuals including sex, total number of relocations and total days of tracking.

capture site	capture year	individual	sex	# locations	tracking duration	present in model
Netherlands	2018	5512398	male	47005	232 days	✓
		5512399	male	53279	238 days	✓
		5512400	female	58462	893 days	✓
		5512401	male	1326	74 days	
		5512402	female	16498	142 days	✓
		5512403	female	68218	294 days	
		5512404	male	78	11 days	
		5512406	female	15615	103 days	✓
		5512407	female	40676	197 days	✓
		5512408	male	553	63 days	
		5512409	male	10287	92 days	✓
		5512410	male	17489	115 days	✓
		5512413	male	335	30 days	
		5512414	male	45390	188 days	✓
		5512415	male	7618	970 days	
		5512416	male	906	62 days	
		5512417	male	68656	297 days	✓
		5512419	female	1	0 days	
		5512420	male	27454	468 days	✓
		5512421	male	64992	499 days	✓
Netherlands	2019	5514520	female	24015	96 days	✓
		5514521	female	34919	98 days	✓
		5514524	female	120774	464 days	✓
		5514527	female	191551	808 days	✓
		5514530	female	16327	78 days	✓
Lithuania	2018	PP00420	male	38030	112 days	
		PP00422	female	48515	559 days	✓
		PP00423	male	58816	349 days	✓
		PP00451	male	60070	887 days	✓
		PP00452	male	49953	153 days	✓
		PP00453	male	32904	201 days	
		PP00454	male	116416	805 days	✓
		PP00455	female	14591	41 days	✓
		PP00456	female	30606	480 days	✓
Lithuania	2019	PP00441	male	33963	375 days	✓
		PP00442	male	3224	9 days	
		PP00443	male	22285	792 days	✓
		PP00444	male	119181	789 days	✓
		PP00445	male	50383	452 days	✓

Table S 2: The tags were programmed according to the following schedule, with GPS-bursts referring to the collection of 15s of 1Hz GPS-data when indicated. Throughout, we used the Ornitela OrniTrack-15 GPS/GSM transmitters for the study.

**Battery level**	**Sampling frequency**	**GPS-bursts**	**Data transmission interval**
$> 75\%$	1 h^{-1}	yes	12 h^{-1}
$> 50\%$ & $<75\%$	2 h^{-1}	yes	12 h^{-1}
$> 25\%$ & $<50\%$	6 h^{-1}	no	12 h^{-1}
$< 25\%$	24 h^{-1}	no	12 h^{-1}

Table S 3: Below we detail the estimates with 95% confidence intervals and model statistic for the fixed effects included in the model for wigeon arrival timing relative to the growing degree days accumulated after the TGS_{onset} (see Table 2 for the respective results for $\Delta\text{arrival}_{\text{d}}$). The model had a residual deviance of 0.088 (null deviance: 0.992; residual deviance of conditional model only: 0.214), and the fixed effects contributed to a marginal $R^2 = 0.29$. The response and independent model terms were scaled and centered, and the model was fitted using 206 arrival events for 27 wigeons and three years. More details on the conditional model, including the random effect term and the correlation structure can be found in Appendix 3.

	Estimate	95% CI	z-value
Intercept (2018)	0.60	0.13 – 1.08	2.52
$TGS_{\text{deviation}}$	-0.01	-0.07 – 0.04	-0.48
Maximum longitude	-0.51	-0.86 – -0.17	-2.93
Max. longitude : Distance traveled	-0.27	-0.36 - -0.18	-6.19
Observation year 2019	-0.33	-1.11 - 0.46	-0.82
Observation year 2020	-0.21	-1.73 - 1.31	-0.27

Table S 4: Below we detail the estimates with 95% confidence intervals and model statistic for the fixed effects included in the model for wigeon arrival timing relative to the growing degree days accumulated after the TGS_{onset} using only the last arrivals for each individual and year. The fixed effects contributed to a marginal $R^2 = 0.31X$. The response and independent model terms were scaled and centered, and the model was fitted using 31 arrival events for 27 wigeons and three years.

	Estimate	95% CI	z-value
Intercept	0.91	0.40 – 1.42	3.48
$TGS_{\text{deviation}}$	-0.16	-0.60 – 0.27	-0.74
Maximum longitude	-0.78	-1.21 – -0.35	-3.53

Supplementary Figures

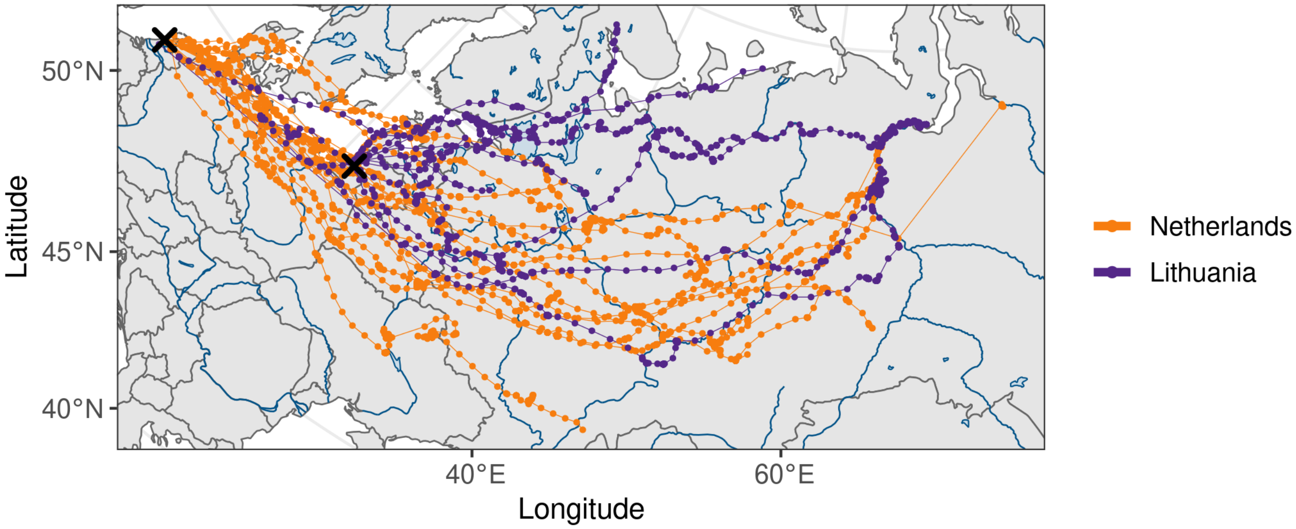

Figure S 1: **Spring migratory movements of Eurasian wigeons.** The map shows the spring migratory tracks of wigeons. Individuals caught in the Netherlands are shown in orange, and wigeons caught in the Nemunas delta in Lithuania in purple. The capture locations are marked with black crosses. Data are shown in a custom two-point equidistant projection.

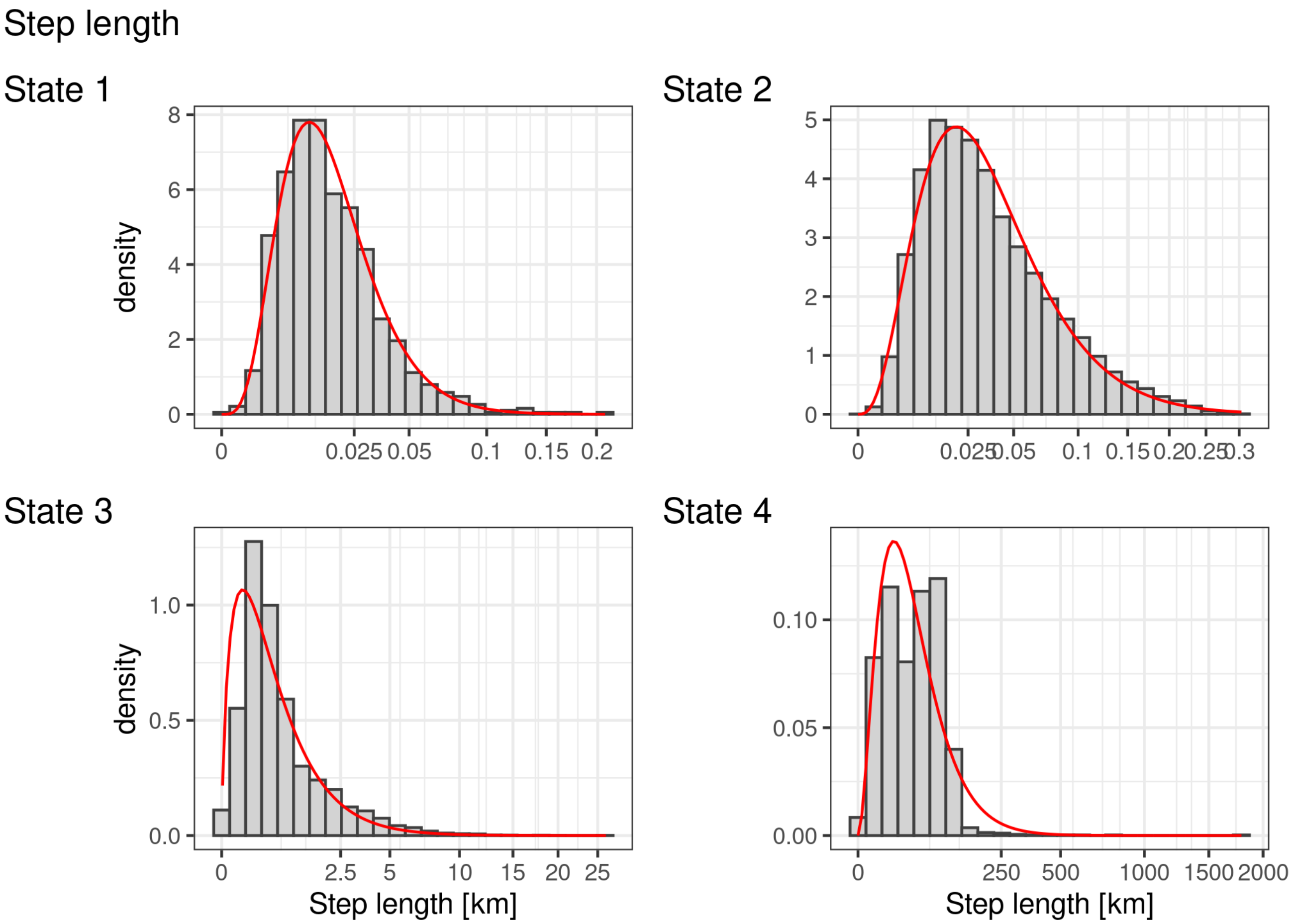

Figure S 2: **Fitted distributions for step lengths for each movement states.** Here we show histogram for step lengths observed in the tracking data, split by the most likely movement state, specifically a) resting, b) non-flight, c) local movement, and d) migratory movement, of each observation as assigned by the Viterbi algorithm. Lines show the density curves for the respective Gamma distributions for each state. Please note that the x-axis has been square-root transformed.

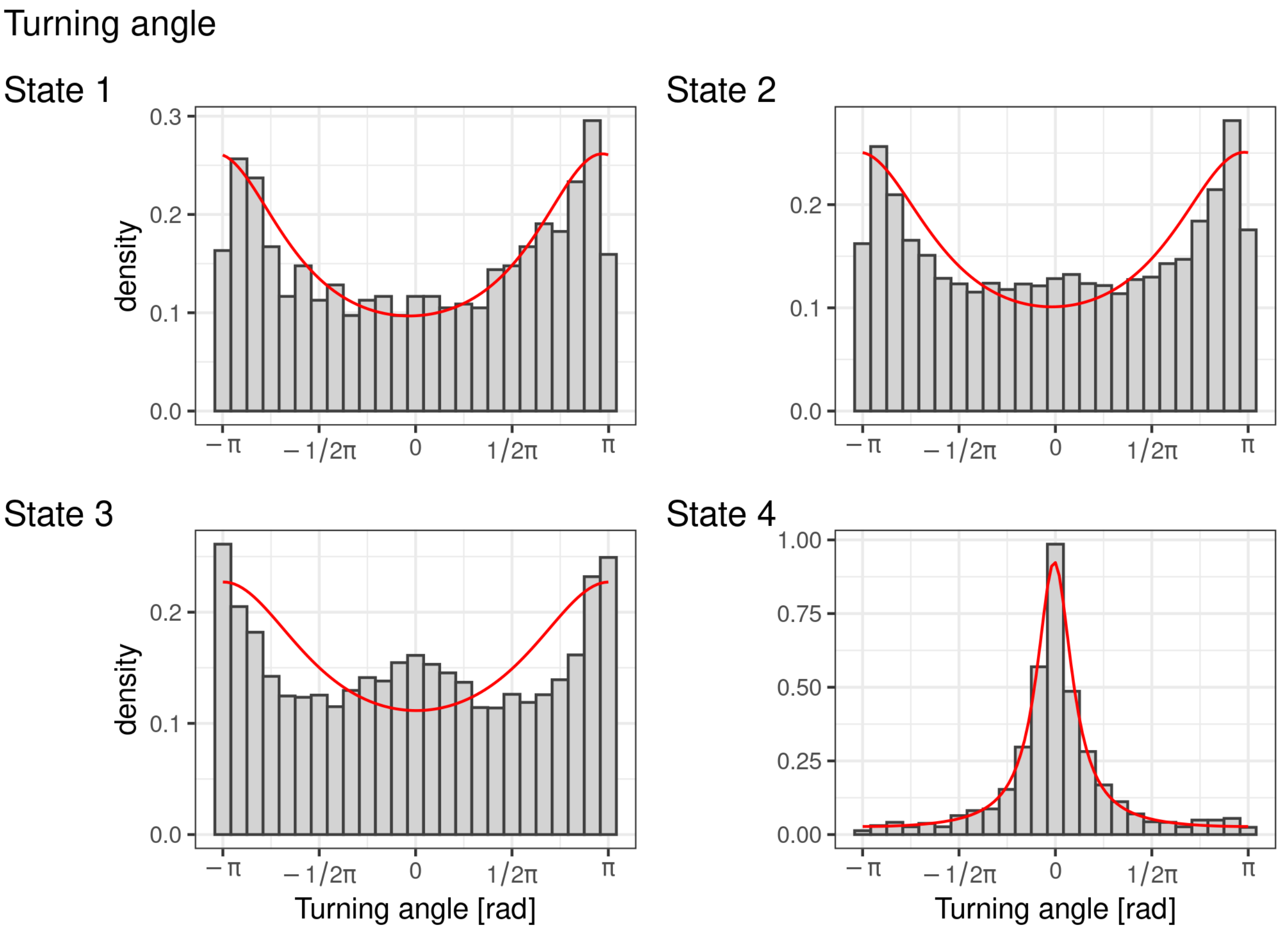

Figure S 3: **Fitted distributions for turning angles for each movement states.** Here we show histogram for turning angles observed in the tracking data, split by the most likely movement state, specifically a) resting, b) non-flight, c) local movement, and d) migratory movement, of each observation as assigned by the Viterbi algorithm. Lines show the density curves for the respective wrapped Cauchy distributions for each state.

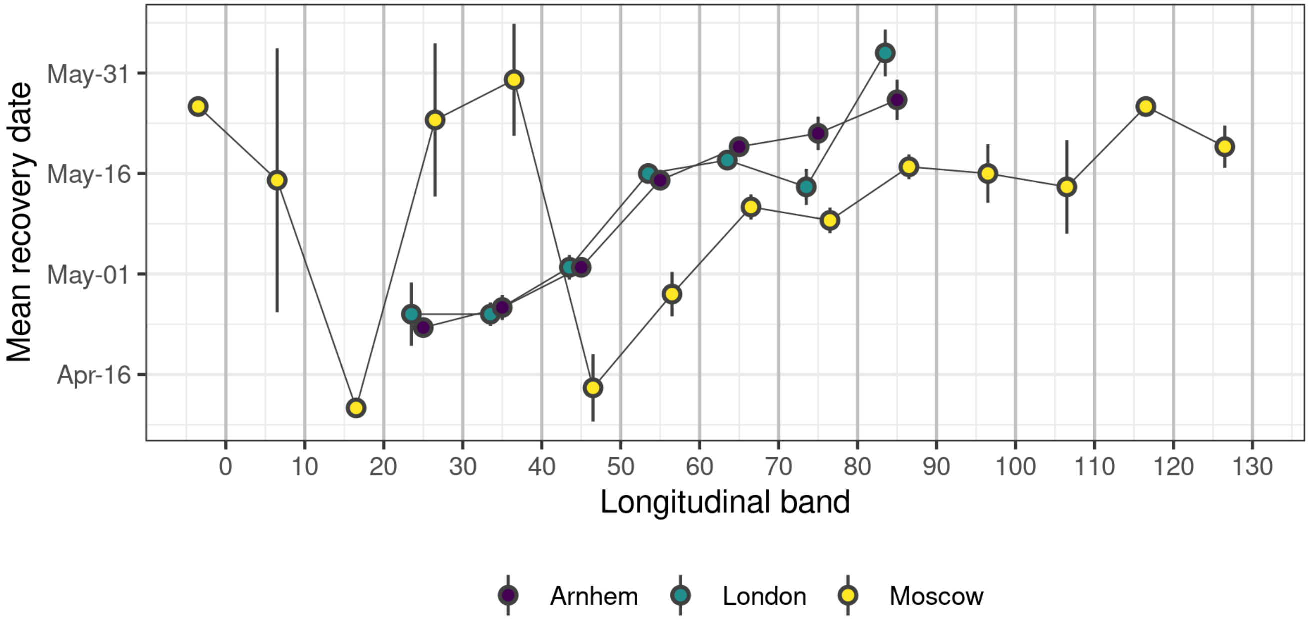

Figure S 4: **Mean recovery dates for ring recovery data**. Shown are the mean and s.d. of recovery dates for ring recoveries in each of the longitudinal bands. The recovery dates are shown separately for the ring recovery schemes of Arnhem, London, and Moscow. Note that we here show the entire range of the ring recovry data, whereas the comparison with tracked wigeons only covered the spatial extent of tracked wigeon locations, from 0 to 80 °E.

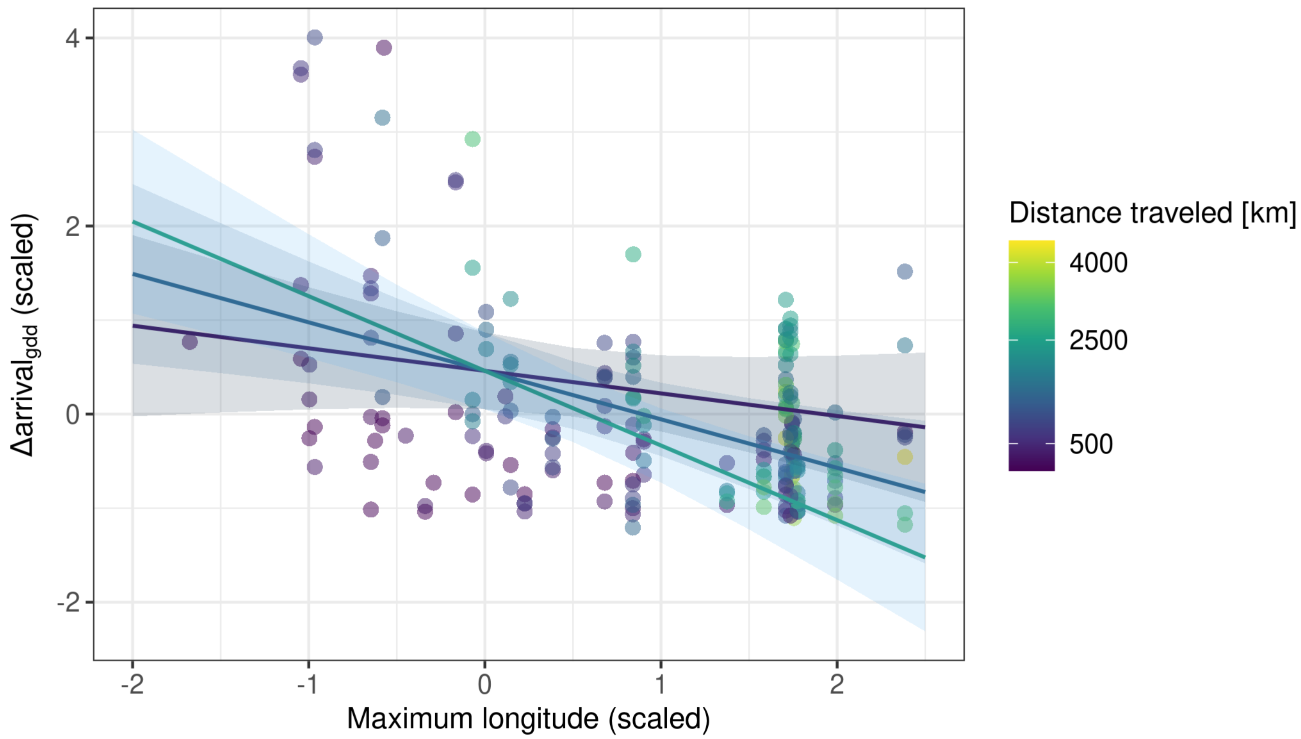

Figure S 5: **Wigeon arrival timing relative to the $TGS_{\mathbf{onset}}$.** Here we show the interaction effect between maximum longitude and distance traveled on growing degree days accumulated after the TGS_{onset}, speficially the effect of maximum longitude for 25%, 50%, and 75% quantile of distance traveled. The lines show the estimate for the effect at the given distance, and the shaded areas reflect the 95% confidence intervals for the estimate. The data used to fit the model are shown as scatterplot.

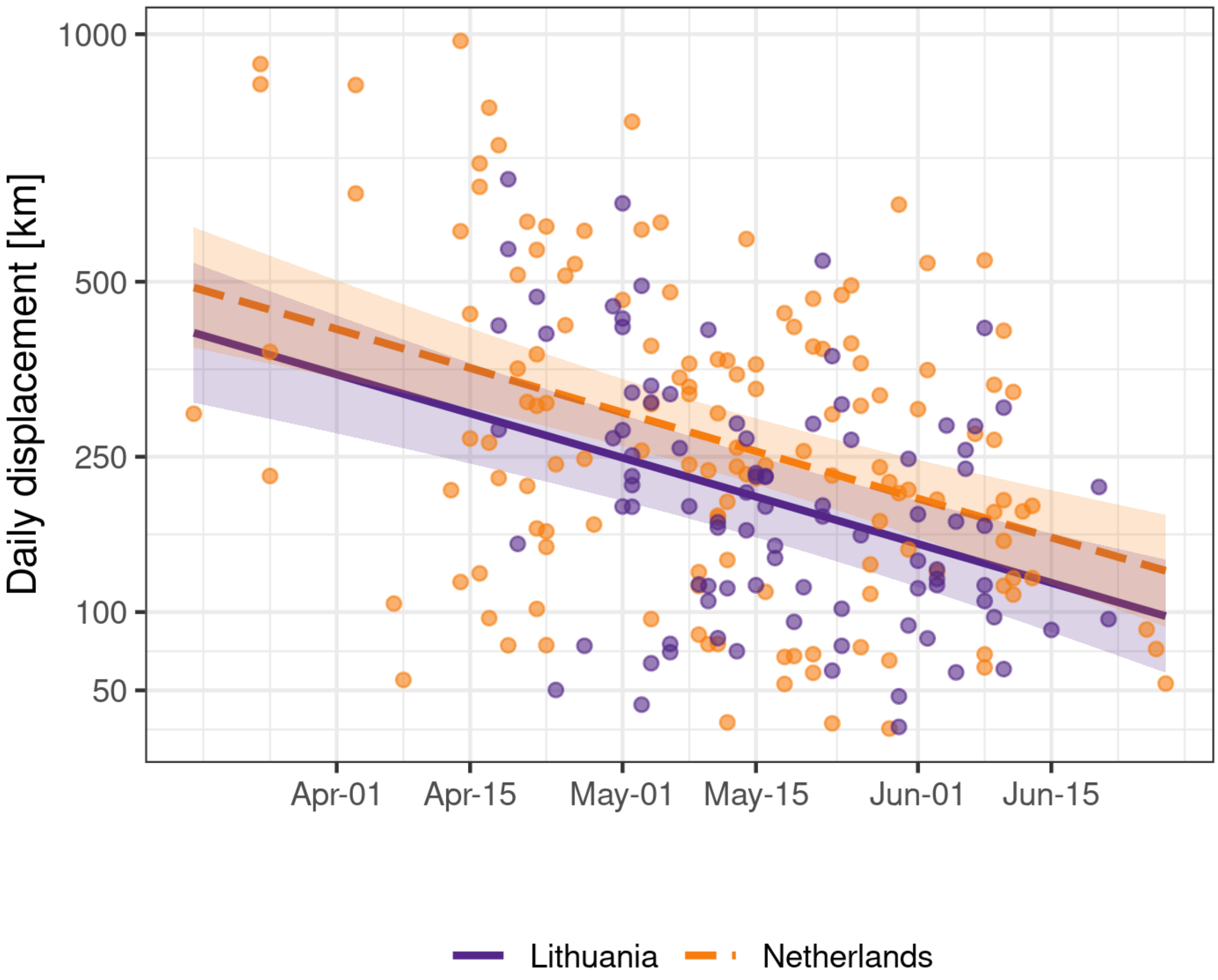

Figure S 6: **Daily displacement distances during active migration.** The scatterplot shows daily displacement distances of wigeons over the course of spring. Note that only days of active migration were included in this figure. The colours highlight the site of capture.
