# Supplementary material for: Migration distance affects how closely Eurasian wigeons follow spring phenology during migration
Source: Mov Ecol. 2021 Dec 11;9:61. doi: 10.1186/s40462-021-00296-0 (PMC8665524; doi:10.1186/s40462-021-00296-0)
Supplement: Supplementary file 2 — Additional file 2. Analysis of wigeon arrival timing (R-code) [file 40462_2021_296_MOESM2_ESM.html]

Additional file 2: Analysis of wigeon arrival timing during spring migration


# Additional file 2: Analysis of wigeon arrival timing during spring migration

#### Mariëlle van Toor

#### 7/30/2021

- Session set-up
- Import & cleaning of tracking data
  - Data import
  - Removing duplicate events
  - Filtering inactive locations at tail end of tracks
- Data preparation for Hidden Markov model
  - Regularising the tracking data
  - Restricting tracks to period relevant for spring migration
  - Calculating solar time at observations
- A hidden Markov model for wigeon spring migration
  - Formatting of tracking data
  - Determining starting parameters for the HMM
  - Fitting a Hidden Markov model
- Identifying arrival events
- Annotation of arrival events with environmental information:
  - Convex hull of wigeon tracking data
  - Import and preparation of environmental data
  - Annotation of arrival events with TGS\_onset and TGS\_deviation
  - Annotation of arrival events GDD accumulated between TGS\_onset and arrival
  - Add distance traveled
  - Scaling of predictors
  - Determine maximum longitude reached by individuals
  - Summary of arrival events
- Models for wigeon arrival timing at staging sites
  - Taking into account repeated observations / longitudinal data
  - Models for arrival conditions
  - Repeating models with only the last arrival for each individual & year

## Session set-up

Import of relevant packages et al.

```
# spatial / temporal
library(rgdal) # link to GDAL library
library(lubridate) # time operations
library(geosphere) # spherical trigonometry
library(maptools) # package providing functionality for calculating solar noon
library(raster)
library(rgeos)

# fitting Hidden Markov Models
library(momentuHMM)

# plotting & misc
library(ggplot2)
library(viridisLite)
library(plyr)
library(patchwork)
library(sf)
library(wesanderson)
library(interactions)

# regression models
library(glmmTMB)
library(sjPlot)
library(DHARMa)

# projection statements used in the analysis
proj.ll <- CRS('+proj=longlat +datum=WGS84')

# wigeon capture sites
capture.sites <- data.frame(site=c('Netherlands', 'Lithuania'), 
                            long=c(4.768945, 21.467045), 
                            lat=c(52.527679, 55.266407), stringsAsFactors=FALSE)

Sys.setlocale("LC_ALL","en_US.UTF-8")
```

```
## R version 4.1.2 (2021-11-01)
## Platform: x86_64-pc-linux-gnu (64-bit)
## Running under: Ubuntu 20.04.3 LTS
## 
## Matrix products: default
## BLAS:   /usr/lib/x86_64-linux-gnu/blas/libblas.so.3.9.0
## LAPACK: /usr/lib/x86_64-linux-gnu/lapack/liblapack.so.3.9.0
## 
## locale:
##  [1] LC_CTYPE=en_US.UTF-8       LC_NUMERIC=C              
##  [3] LC_TIME=en_US.UTF-8        LC_COLLATE=en_US.UTF-8    
##  [5] LC_MONETARY=en_US.UTF-8    LC_MESSAGES=en_US.UTF-8   
##  [7] LC_PAPER=sv_SE.UTF-8       LC_NAME=C                 
##  [9] LC_ADDRESS=C               LC_TELEPHONE=C            
## [11] LC_MEASUREMENT=sv_SE.UTF-8 LC_IDENTIFICATION=C       
## 
## attached base packages:
## [1] stats     graphics  grDevices utils     datasets  methods   base     
## 
## other attached packages:
##  [1] effects_4.2-0      carData_3.0-4      merTools_0.5.2     arm_1.11-2        
##  [5] MASS_7.3-54        DHARMa_0.4.3       sjPlot_2.8.9       glmmTMB_1.1.2     
##  [9] lme4_1.1-27.1      Matrix_1.3-4       interactions_1.1.5 wesanderson_0.3.6 
## [13] sf_1.0-1           patchwork_1.1.1    plyr_1.8.6         viridisLite_0.4.0 
## [17] ggplot2_3.3.5      momentuHMM_1.5.3   rgeos_0.5-5        raster_3.4-13     
## [21] maptools_1.1-1     geosphere_1.5-10   lubridate_1.7.10   rgdal_1.5-23      
## [25] sp_1.4-5          
## 
## loaded via a namespace (and not attached):
##   [1] backports_1.2.1     blme_1.0-5          jtools_2.1.3       
##   [4] Hmisc_4.5-0         TMB_1.7.21          splines_4.1.2      
##   [7] TH.data_1.0-10      digest_0.6.27       foreach_1.5.1      
##  [10] htmltools_0.5.1.1   fansi_0.5.0         magrittr_2.0.1     
##  [13] checkmate_2.0.0     cluster_2.1.2       doParallel_1.0.16  
##  [16] crawl_2.2.1         modelr_0.1.8        sandwich_3.0-1     
##  [19] CircStats_0.2-6     jpeg_0.1-8.1        colorspace_2.0-2   
##  [22] mitools_2.4         xfun_0.24           dplyr_1.0.7        
##  [25] crayon_1.4.1        jsonlite_1.7.2      survival_3.2-13    
##  [28] zoo_1.8-9           iterators_1.0.13    glue_1.4.2         
##  [31] gtable_0.3.0        emmeans_1.6.2-1     sjstats_0.18.1     
##  [34] sjmisc_2.8.7        abind_1.4-5         scales_1.1.1       
##  [37] mvtnorm_1.1-2       DBI_1.1.1           rngtools_1.5       
##  [40] ggeffects_1.1.0     Rcpp_1.0.7          xtable_1.8-4       
##  [43] performance_0.7.2   htmlTable_2.2.1     units_0.7-2        
##  [46] foreign_0.8-81      proxy_0.4-26        Formula_1.2-4      
##  [49] survey_4.1-1        htmlwidgets_1.5.3   datawizard_0.1.0   
##  [52] RColorBrewer_1.1-2  ellipsis_0.3.2      pkgconfig_2.0.3    
##  [55] nnet_7.3-16         sass_0.4.0          utf8_1.2.1         
##  [58] tidyselect_1.1.1    rlang_0.4.11        later_1.2.0        
##  [61] effectsize_0.4.5    munsell_0.5.0       tools_4.1.2        
##  [64] cli_3.0.1           generics_0.1.0      sjlabelled_1.1.8   
##  [67] broom_0.7.8         evaluate_0.14       stringr_1.4.0      
##  [70] fastmap_1.1.0       yaml_2.2.1          knitr_1.33         
##  [73] pander_0.6.4        purrr_0.3.4         nlme_3.1-152       
##  [76] doRNG_1.8.2         mime_0.11           compiler_4.1.2     
##  [79] rstudioapi_0.13     png_0.1-7           e1071_1.7-7        
##  [82] tibble_3.1.2        bslib_0.2.5.1       stringi_1.7.3      
##  [85] parameters_0.14.0   Brobdingnag_1.2-6   lattice_0.20-45    
##  [88] classInt_0.4-3      nloptr_1.2.2.2      vctrs_0.3.8        
##  [91] pillar_1.6.1        lifecycle_1.0.0     jquerylib_0.1.4    
##  [94] estimability_1.3    data.table_1.14.0   insight_0.14.2     
##  [97] httpuv_1.6.1        R6_2.5.0            latticeExtra_0.6-29
## [100] promises_1.2.0.1    KernSmooth_2.23-20  gridExtra_2.3      
## [103] codetools_0.2-18    boot_1.3-28         assertthat_0.2.1   
## [106] withr_2.4.2         broom.mixed_0.2.7   multcomp_1.4-17    
## [109] bayestestR_0.10.0   parallel_4.1.2      grid_4.1.2         
## [112] rpart_4.1-15        tidyr_1.1.3         coda_0.19-4        
## [115] class_7.3-19        minqa_1.2.4         rmarkdown_2.9      
## [118] numDeriv_2016.8-1.1 shiny_1.6.0         base64enc_0.1-3
```

## Import & cleaning of tracking data

The data are stored on Movebank in four different studies, one for each name and study site. Here, I import all for data sets and merge them into a single data.frame, and attach the sex of individuals using the reference data also downloaded from Movebank. Furthermore, I filter the data to be from wigeons only, since one of the studies contains data from a single pintail that was caught in the same net as the wigeons.

### Data import

```
fil <- list.files('data/tracking_data', full.names=T)
track.files <- fil[!grepl('reference', fil)]
inf.files <- fil[grepl('reference', fil)]

tracks <- rbind.fill(lapply(track.files, function(f){
  print(f)
  tmp <- read.delim(f, header=T, sep=',', as.is=T)
  tmp$timestamp <- as.POSIXct(tmp$timestamp, tz='UTC')
  tmp$site <- ifelse(grepl('Netherlands', f), 'Netherlands', 'Lithuania')
  year <- gsub(' \\(.*', '', gsub('.csv', '', f))
  tmp$year <- gsub(' ', '', (rev(strsplit(year, ' ')[[1]])[1]))
  
  inf <- read.delim(inf.files[gsub('-ref.*', '', inf.files) %in% gsub('.csv', '', f)], header=T, sep=',', as.is=T)
  inf <- inf[!is.na(inf$animal.id),]
  
  tmp <- ddply(tmp, 'individual.local.identifier', function(x){
    id <- unique(x$individual.local.identifier); #print(id)
    y <- inf[inf$animal.id==id,]
    x$sex <- y$animal.sex
    return(x)
  })
}))
```

```
## [1] "data/tracking_data/Dabbling duck migration Lithuania 2019.csv"
## [1] "data/tracking_data/Eurasian wigeons spring 2018 (Lithuania).csv"
## [1] "data/tracking_data/Eurasian wigeons spring 2018 (Netherlands).csv"
## [1] "data/tracking_data/Eurasian wigeons spring 2019 (Netherlands).csv"
```

```
# only retain wigeons
tracks <- tracks[tracks$individual.taxon.canonical.name=='Anas penelope',]
```

### Removing duplicate events

The Ornitela tags we used in this study send the data via GPRS whenever possible. If GPRS connection cannot be achieved, the tags instead send a SMS with recent coordinates but without additional sensor information. As soon as GSM becomes available again, these location are re-sent in full, and consequently it can happen that duplicate entries might be present in the data set. Here, I will identify duplicate events for each individuals and use the transmission protocol (“GPRS” or “SMS” in the column “orn.transmission.protocol”) to remove those duplicates that were sent via SMS.

```
# are there any duplicated events?
table(sapply(split(tracks, tracks$individual.local.identifier), function(x){any(duplicated(x$timestamp))}))
```

```
## 
## FALSE  TRUE 
##    32     7
```

```
# 7 individuals have duplicate timestamps

# this can happen if location data are initially sent via SMS istead of GPRS
table(tracks$orn.transmission.protocol)
```

```
## 
##    GPRS     SMS 
## 1611234     127
```

```
tracks <- ddply(tracks, 'individual.local.identifier', function(tmp){
  if(any(duplicated(tmp$timestamp))){
    dup.time <- tmp$timestamp[duplicated(tmp$timestamp)]
    for(t in dup.time){
      check <- which(tmp$timestamp==t)
      check.trans <- tmp$orn.transmission.protocol[check]
      if(any(check.trans=='SMS')){
        tmp <- tmp[-check[check.trans=='SMS'],]
      }
    }
    return(tmp)
  }else{
    return(tmp)
  }
})

# this was sufficient to remove all duplicate timestamps
# no other actions required!
table(sapply(split(tracks, tracks$individual.local.identifier), function(x){any(duplicated(x$timestamp))}))
```

```
## 
## FALSE 
##    39
```

### Filtering inactive locations at tail end of tracks

It can happen that individuals get predated, or in the case of ducks shot by hunters, or even loose their tags. In either case, the tag might still be active while not attached to a live animal. This occurred to several of our individuals. To make sure that we only include locations collected while tags were attached to live individuals, we here remove inactive locations at the tail end of each trajectory. Here, we define “inactive” when instantaneous ground speed as measured by the tags equals exactly 0.

```
nrow(tracks)
```

```
## [1] 1611248
```

```
# remove inactive locations at the tail of data
tracks <- ddply(tracks, 'individual.local.identifier', function(x){
  check <- rev(x$ground.speed)[1]==0
  while(check & nrow(x)>0){
    x <- x[-nrow(x),]
    check <- rev(x$ground.speed)[1]==0
  }
  return(x)
})

nrow(tracks)
```

```
## [1] 1610746
```

## Data preparation for Hidden Markov model

### Regularising the tracking data

The data that we collect are usually bursted - if the battery levels allow, the tags will record multiple locations in short succession (1Hz) every time the tag is scheduled to record a fix, rather than just a single one. To be able to develop a hidden Markov model for these data, the data do however need to be (near-)regular - meaning we need to “remove” bursts. Ideally, this should be easy by just looking at the time difference between subsequent locations. However, in reality it often takes more time than scheduled to acquire GPS positions, and consequently the time difference between subsequent locations differs from the scheduled ideal. However, the Ornitela tags do provide an estimate of the time to acquire the GPS position, meaning we can infer when a fix was scheduled to be taken. Since the coarse schedule (locations sampled once per hour, or less frequently depending on battery level) was very different from the burst schedule (sampling frequency 1/s), we can do this by calculating the time lag between subsequent locations. Since the bursts were programmed to sample 15 locations, we will use a maximum lag of 15 locations:´

```
tracks$obs.year <- year(tracks$timestamp)
tracks$obs.month <- month(tracks$timestamp)

#tracks <- tracks[tracks$obs.month %in% 2:8,]

tracks$individual.year <- paste(tracks$individual.local.identifier, tracks$obs.year, sep='-')
tracks <- tracks[tracks$obs.year<2021,]

# code below only works if we have at least 15 locations for an individual
check <- ddply(tracks, 'individual.year', nrow)
tracks <- tracks[tracks$individual.year %in% check$individual.year[check$V1>15],]

tracks <- ddply(tracks, 'individual.year', function(x){
  #print(unique(x$individual.year))
  burst.length <- 15; cutoff.lag <- 3000
  
  timelags <- as.data.frame(lapply(1:(burst.length-1), function(l){
    c(rep(NA, l), diff(as.numeric(x$timestamp), lag=l))}))
  names(timelags) <- paste0('lag', sprintf('%.2d', 1:length(timelags)))
  
  check <- apply(timelags, 1, function(z){all(z>cutoff.lag, na.rm=T)})
  check[1] <- F
  
  x$burst <- c(0, cumsum(diff(check)>0)) + 1
  
  x <- ddply(x, 'burst', function(y){
    y$dur.burst <- as.numeric(max(y$timestamp)) - as.numeric(min(y$timestamp))
    y$n.burst <- nrow(y)
    return(y)
  })
  
  return(x)
})

tracks <- tracks[!duplicated(tracks[,c('individual.year', 'burst')]),]
```

### Restricting tracks to period relevant for spring migration

Since we are interested in spring migration, we will limit the tracks to include this period only. Rather than using a temporal cut-off, I will use the extent of the migratory trajectories to determine reference points for the likely starting and end locations of migration. For any individual, I will treat the southwestern-most location it was observed in during March/April as starting location, and the northeastern-most location it ws observed in during May and June as end location for spring migration.

To limit the tracks to spring migration, I will: - calculate the distance of each location to the respective start and end location - determine the day when the individual was last within a threshold distance from the starting location - determine the day when the individual was first within a threshold distance to the end location - threshold distance will be determined as the distance a wigeon with mean reported airspeed of migratory wigeons - 3 s.d. would cover in one hour (from Pennycuick *et al.* 2013)

Note that we cannot determine the biological function for all of the starting/end sites for certain - starting locations are the supposed wintering locations for individuals caught in the Netherlands, and presumed staging site for individuals captured in Lithuania (in the first year of observation), whereas end locations can include site of predation/loss of contact, breeding location, or post-breeding movement.

```
# from Pennycuick et al. 2013
mean.airspeed <- 18.5
sd.airspeed <- 2.28

# determine threshold distance
threshold.v <- (mean.airspeed-2*sd.airspeed)
threshold.d <- threshold.v * 3.6

# check duration, length of track per year, and whether individuals ever surpassed the threshold distance
check <- ddply(tracks, 'individual.year', function(x){
  d <- distGeo(x[,c('location.long', 'location.lat')])/1000
  t <- (max(as.numeric(x$timestamp)) - min(as.numeric(x$timestamp)))/86400
  data.frame(nrow=nrow(x), duration=round(t), max.dist=round(max(d, na.rm=T)), max.speed=max(x$ground.speed))
})

# keep individuals with max distance > threshold & max speed > equivalent speed
keep <- check$individual.year[check$max.speed>threshold.v]
tracks <- tracks[tracks$individual.year %in% keep,]

# determine starting location (southwestern-most location in March-April)
start.loc <- ddply(tracks, 'individual.year', function(x){
  x <- x[month(x$timestamp) %in% 2:4,]
  
  if(nrow(x)>0){
    proj.tpq <- CRS(paste0("+proj=tpeqd +lat_1=", min(x$location.lat), " +lon_1=", min(x$location.long),
                       " +lat_2=", max(x$location.lat), " +lon_2=", max(x$location.long), 
                       " +x_0=0 +y_0=0 +datum=WGS84 +units=m +no_defs +ellps=WGS84 +towgs84=0,0,0"))
    p <- as.data.frame(spTransform(SpatialPoints(x[,c('location.long', 'location.lat')],
            proj4string=proj.ll), CRSobj=proj.tpq))
  
    sw <- x[which.min(p$location.long),c('location.long', 'location.lat')]
    names(sw) <- c('start.long', 'start.lat')
    return(sw)
  }else{
    return(data.frame(start.long=NA, start.lat=NA))
  }
})

# end location: north-eastern most location recorded during May - July
end.loc <-  ddply(tracks, 'individual.year', function(x){
  x <- x[month(x$timestamp) %in% 5:7,]
  
  if(nrow(x)>0){
    proj.tpq <- CRS(paste0("+proj=tpeqd +lat_1=", min(x$location.lat), " +lon_1=", min(x$location.long),
                       " +lat_2=", max(x$location.lat), " +lon_2=", max(x$location.long), 
                       " +x_0=0 +y_0=0 +datum=WGS84 +units=m +no_defs +ellps=WGS84 +towgs84=0,0,0"))
    p <- as.data.frame(spTransform(SpatialPoints(x[,c('location.long', 'location.lat')],
            proj4string=proj.ll), CRSobj=proj.tpq))
  
    ne <- x[which.max(p$location.long),c('location.long', 'location.lat')]
    names(ne) <- c('end.long', 'end.lat')
    return(ne)
  }else{
    return(data.frame(end.long=NA, end.lat=NA))
  }
})

end.locations <- merge(start.loc, end.loc)
end.locations <- end.locations[complete.cases(end.locations),]
end.locations$distance <- unlist(lapply(1:nrow(end.locations), function(j){
  distGeo(end.locations[j,c('start.long', 'start.lat')], end.locations[j,c('end.long', 'end.lat')])
}))/1000

end.locations <- end.locations[end.locations$distance>threshold.d,]
tracks <- tracks[tracks$individual.year %in% end.locations$individual.year,]

save(end.locations, file='data/migration_endpoints.RData')
```

Now we can determine when individuals leave the starting location, and reach the end location, and cut the tracks to that extent:

```
tracks <- ddply(tracks, 'individual.year', function(x){
  id <- unique(x$individual.year); 
  p.start <- end.locations[end.locations$individual.year==id,c('start.long', 'start.lat')]
  p.end <- end.locations[end.locations$individual.year==id,c('end.long', 'end.lat')]
  
  if(nrow(p.start)==1 & nrow(p.end)==1){
    x$d2start <- distGeo(p.start, x[,c('location.long', 'location.lat')])/1000
    x$d2end <-   distGeo(p.end, x[,c('location.long', 'location.lat')])/1000
    
    start.day <- max(as.Date(x$timestamp[x$d2start<threshold.d]))
    end.day <- min(as.Date(x$timestamp[x$d2end<threshold.d]))
    
    x$migratory <- ifelse(as.Date(x$timestamp) %in% start.day:end.day, T, F)
    
    return(x)  
  }else{
    return(NULL)
  }
})
```

A second factor that comes into play are missed fixes. These can occur when the tag is unable to establish connection with sufficient GPS satellites. Practically, whenever the sampling rate is reduced because battery levels drop, the fixes that are not taken because of lower battery levels can be considered missed fixes as well. While Hidden Markov models require regular data, they can deal with some missed fixes, meaning these do not necessarily make the fitting of HMMs impossible - we’ll be able to run the HMMs even in the light of certain amounts of irregular data. That does however mean that we will have to add these “missed fixes” manually. Here, I will use a function that computes an idealised time series with perfectly regular timestamps. This function then identifies the location that is closest in time, and determines whether it can be considered near-regular or not given a user-supplied threshold value. If no location is considered as sufficiently close to a timestamp in the ideal time series, the location will replaced by a missed fix, i.e. no information about location or other sensory information. Here is the function I wrote to do this:

```
regularise.tracks <- function(tmp, target.time, wiggle.time=NULL){
  
   # if no wiggle room is defined, use 5% of the target sampling frequency
  if(is.null(wiggle.time)){
    wiggle.time <- 0.05*target.time
  }

  # calculate time difference between subsequent locations (in seconds)
  dtime <- unlist(lapply(2:nrow(tmp), function(i){
    as.numeric(difftime(tmp$timestamp[i], tmp$timestamp[i-1], units='secs'))
  }))
  dtime <- c(dtime[1], dtime)
  
  start <- tmp$timestamp[1]
  if(is.na(start)){return(NULL)}
  
  # match target timestamps (regular series) with observed timeseries of observations; forward
  get.closest.forward <- seq(start, max(tmp$timestamp), by=target.time)
  closest <- rbind.fill(lapply(get.closest.forward, function(t){
    check <- abs(as.numeric(difftime(tmp$timestamp, t, unit='secs')))
    data.frame(target=t, timestamp=tmp$timestamp[which.min(check)], diff=min(check))
  }))

  # calculate time difference between subsequent observations (just the closest matches to the idealised timeseries)
  closest$t.prev <- c(NA, as.numeric(diff(closest$timestamp, units='secs'), units='secs'))
  closest$diff.prev <- abs(target.time-closest$t.prev)

  # merge ideal and observed time series
  tmp <- merge(tmp, closest, by='timestamp', all.y=T)
  tmp$check <- tmp$diff<=wiggle.time|tmp$diff.prev<=wiggle.time
  
  return(tmp)
}
```

Now we can apply the function to the wigeon trajectories, separately to each individual and year of observation. I will use 600 seconds / 10 minutes of wiggle time for a sampling frequency of 1 per hour.

```
# one individual collected locations at a different schedule (every two hours instead of every hour)
# those will be regularised using a two-hourly schedule instead

tracks <- ddply(tracks, c('individual.year'), function(tmp){
#  print(unique(tmp$ID)); print(nrow(tmp))
  id <- unique(tmp$individual.year)
  if(id=='PP00456-2018'){
    tmp <- regularise.tracks(tmp, target.time=7200, wiggle.time=600)
  }else{
    tmp <- regularise.tracks(tmp, target.time=3600, wiggle.time=600)
  }
  
  return(tmp)
})

# how many locations are considered regular?
table(tracks$check)
```

```
## 
## FALSE  TRUE 
## 45453 92920
```

Let’s inspect the results:

We can see here that for many of the individuals, consistently near-regular data are available with small exceptions. These will work well for the intended analyses, though we might have to remove sections or even entire individuals’ tracks if insufficient data are available.

Now we can also assess whether the tracks of the remaining individuals are sufficiently regular, and of sufficient duration to be of interest. Here I will first apply a simple segmentation into regular and irregular bits of tracks, and lump segments whenever the duration of irregular sampling between regular segments is <24 hours. Then we can assess how much data (in terms of duration) individuals can provide for the analysis by calculating the duration of the longest section of nearly uninterrupted, nearly regular data for each individual:

```
wigeon.tracks <- ddply(tracks[tracks$check,], 'individual.year', function(tmp){
  id <- unique(tmp$individual.year)
  tmp$segment <- c(0, cumsum(diff(tmp$check)!=0)) + 1
  tmp$ID <- paste(id, sprintf('%.2d', tmp$segment), sep='-')

  # lump regular segments if less than 12 hours apart
  seg.dur <- ddply(tmp, 'segment', function(x){
    dur <- as.numeric(difftime(max(x$timestamp), min(x$timestamp), units='secs'))
    class <- ifelse(all(x$check), 'regular', 'irregular')
    data.frame(duration=dur, type=class)
  })
  
  threshold <- 36*3600
  seg.dur$inrease.seg <- ifelse(seg.dur$type=='regular'|seg.dur$duration<threshold, FALSE, TRUE)
  seg.dur$new.seg <- c(0, cumsum(diff(seg.dur$inrease.seg)!=0)) + 1
  
  # reassign newly defined segments
  tmp <- ddply(tmp, 'segment', function(x){
    x$segment <- seg.dur$new.seg[seg.dur$segment==unique(x$segment)]
    return(x)
  })
  
  return(tmp)
})

# does any individual have more than one segment?
unique(wigeon.tracks$segment)
```

```
## [1] 1
```

```
# no, so we can re-assign ID as individual-year
wigeon.tracks$ID <- wigeon.tracks$individual.year
```

Now we can calculate the duration of the longest segment per individual-year:

```
max.dur <- ddply(wigeon.tracks, 'individual.year', function(tmp){
  dur <- ddply(tmp, 'segment', function(x){
    d <- difftime(max(x$timestamp), min(x$timestamp), unit='days')
    data.frame(duration=as.numeric(d), n.samples=nrow(x))
  })
  return(dur[which.max(dur$duration),])
})

sort(round(max.dur$duration))
```

```
##  [1]  41  64  70  85  94  95  96  98 103 107 115 125 130 137 138 140 142 147 167
## [20] 171 173 187 188 192 192 206 232 237 238 239 273 273 280 291 292
```

What remains if we only consider regular & migratory data?

Sample sizes:

```
wigeon.tracks <- wigeon.tracks[wigeon.tracks$check & wigeon.tracks$migratory,]

summ <- ddply(wigeon.tracks, c('individual.local.identifier', 'obs.year'), function(x){
  d <- diff(as.numeric(range(x$timestamp)))/86400
  data.frame(n.locations=nrow(x), n.days=length(unique(as.Date(x$timestamp))), duration=d)
})
  
print(summ)
```

```
##    individual.local.identifier obs.year n.locations n.days   duration
## 1                      5512398     2018        1811     78 153.166586
## 2                      5512399     2018        1881     80 172.165185
## 3                      5512400     2018        1619     70 143.178715
## 4                      5512400     2019         660     32  37.885058
## 5                      5512402     2018        1003     47 130.919178
## 6                      5512403     2018        1606     70 161.177951
## 7                      5512406     2018         973     43 102.971447
## 8                      5512407     2018         313     15  90.075312
## 9                      5512409     2018         251     13  70.120150
## 10                     5512410     2018        1070     47 113.968495
## 11                     5512414     2018        1504     66 125.970197
## 12                     5512417     2018        2402    103 172.062836
## 13                     5512420     2018        1240     54 120.059687
## 14                     5512421     2018        2278    100 156.075660
## 15                     5514520     2019        1405     61  85.470405
## 16                     5514521     2019        2068     87  86.944757
## 17                     5514524     2019        1335     58  78.476400
## 18                     5514524     2020         951     40  39.950775
## 19                     5514527     2019        1562     67  67.976007
## 20                     5514530     2019         906     40  60.433877
## 21                     PP00420     2018         141      6   5.957303
## 22                     PP00422     2018         379     16  15.963681
## 23                     PP00423     2018         926     39  38.949769
## 24                     PP00441     2019          71      3   2.937940
## 25                     PP00443     2019         238     10   9.957847
## 26                     PP00444     2019        2327     98  97.967106
## 27                     PP00444     2020        1590     80  91.287789
## 28                     PP00445     2019        1305     55  54.958322
## 29                     PP00451     2018        2158     91  90.971354
## 30                     PP00452     2018         381     16  15.992326
## 31                     PP00453     2018         238     10   9.961123
## 32                     PP00454     2018         665     28  27.937500
## 33                     PP00454     2019         354     15  14.973287
## 34                     PP00455     2018         832     35  34.969375
## 35                     PP00456     2018         263     22  21.983218
```

```
length(unique(summ$individual.local.identifier))
```

```
## [1] 31
```

```
rev(sort(table(summ$individual.local.identifier)))
```

```
## 
## PP00454 PP00444 5514524 5512400 PP00456 PP00455 PP00453 PP00452 PP00451 PP00445 
##       2       2       2       2       1       1       1       1       1       1 
## PP00443 PP00441 PP00423 PP00422 PP00420 5514530 5514527 5514521 5514520 5512421 
##       1       1       1       1       1       1       1       1       1       1 
## 5512420 5512417 5512414 5512410 5512409 5512407 5512406 5512403 5512402 5512399 
##       1       1       1       1       1       1       1       1       1       1 
## 5512398 
##       1
```

```
table(summ$obs.year)
```

```
## 
## 2018 2019 2020 
##   22   11    2
```

### Calculating solar time at observations

Given that wigeons seems to mostly initiate migratory movements during the evening, it might be of advantage of any HMM to include time of day as a covariate for state switching probabilities (i.e. the probability of individuals to switch from one movement mode to another). While we have timestamps associated with each locations, these are not directly useful as all timestamps are in UTC - a poor proxy for time of day for wigeons breeding in the Ob River delta. In general the perceived time of day might change with seasons as well, as day length increases as spring progresses. I will remedy part of these complication by calculating solar time (the time relative to noon) at the individuals’ locations, wherever they are. I will subsequently shift this solar tie to reflect fractional hours of the day, ranging from 0 - 24 to make it easier to understand any model results.

```
wigeon.tracks <- ddply(wigeon.tracks, 'individual.local.identifier', function(tmp){
  tmp$solar.time <- unlist(lapply(1:nrow(tmp), function(j){
    if(!is.na(tmp$location.long[j])){
      p <- SpatialPoints(tmp[j,c('location.long', 'location.lat')], proj4string=CRS('+proj=longlat +datum=WGS84'))
    }else{
      index <- (j-12):(j+12)
      index <- index[index>0 & index<=nrow(tmp)]
      x <- mean(tmp$location.long[index], na.rm=T)
      y <- mean(tmp$location.lat[index], na.rm=T)
      p <- SpatialPoints(data.frame(x=x, y=y), proj4string=CRS('+proj=longlat +datum=WGS84'))  
    }
    noon <- solarnoon(p, tmp$timestamp[j], POSIXct.out=TRUE)$time
    tod <- as.numeric(difftime(tmp$timestamp[j], noon, units='hours'))+12
    tod <- tod%%24
    return(tod)
  }))
  return(tmp)
})

# also add consecutive days of the year, with January 01 corresponding to day 1
wigeon.tracks$julian <- as.numeric(strftime(as.character(wigeon.tracks$timestamp), format='%j'))

# regular sampling throughout
hist(wigeon.tracks$solar.time, breaks=23)
```

```
save(wigeon.tracks, file='data/202106_migratory_tracks_regular.RData')
```

## A hidden Markov model for wigeon spring migration

### Formatting of tracking data

During this step, the *prepData()* also calculates step length between subsequent locations, as well as turning angles. In my experience, HMMs are more successful at fitting good distributions for the step lengths of duck migratory movements if we use square-root transformed step length instead. This is an experience I have made for several species, and so too for wigeons. In the process of finding good starting parameters for this particular data set, I have tried both step length and its square-root, and square-root transformed step length consistently produced better results for the intended purpose, which is why it is shown here.

```
load('data/202106_migratory_tracks_regular.RData')

# each individual is required to have at least three observations:
wigeon.tracks <- ddply(wigeon.tracks, 'ID', function(x){
  if(nrow(x)<=3){
    return(NULL)
  }else{
    return(x)
  }
})

wigeon.df <- prepData(wigeon.tracks, type='LL', coordNames=c('location.long', 'location.lat'))
wigeon.df$step.sqrt <- sqrt(wigeon.df$step)
```

### Determining starting parameters for the HMM

Finding a good set of starting parameters can take quite some experimentation and testing different combinations of parameters and number of states. Most often, either a Gamma or Weibull distribution (i.e. members of the exponential distribution family with fat tails) tend to work well for both step lengths and ground speed distributions in ducks. Similarly, a wrapped Cauchy or von Mises distribution tend to work well for turning angles. I tested all of these, and found that for this data, a Weibull distribution works best for wigeon (square-root transformed) step length, and a wrapped Cauchy distribution for turning angles. For starting parameters, it is always good to inspect the data visually, and think about what behaviours one could expect. Here, I am fitting a four-state model with which I am attempting to model four different behaviours:

- **resting**: ducks are asleep or resting on either land or water. Step length should be near-zero (~GPS error), and turning angles should be rather uniform (low concentration around the mean)
- **non-flight**: ducks are moving by either walking (e.g. while foraging) or actively swimming. Consequently, step lengths should be greater than GPS error, but not exceed what could be expected under ground speeds of about 1.5 m/s. Turning angles again are expected to have low concentration.
- **local movement**: ducks actively fly, but only perform local movements, e.g. because they are scared up by a predator, or move to a safe resting area at dawn. Step lengths are clearly larger than local movements, but should be shorter than could be expected from constant direct flight between fixes (~20 m/s x 3600s = ~72 km). Concentration of turning angles should be more concentrated, but concentrate around -pi and pi as we expect back and forth movements rather than directed movement towards a distant goal
- **migratory movement**: clear directed movement at high speed. Here, I used an estimate from the following study to estimate expected step lengths for migration: *Pennycuick, C. J., Åkesson, S., & Hedenström, A. (2013). Air speeds of migrating birds observed by ornithodolite and compared with predictions from flight theory. Journal of the Royal Society Interface, 10(86), 20130419*. Turning angles should be strongly concentrated around 0.

```
n.states <- 4
state.names <- c('rest', 'non-flight', 'local', 'migratory')

# wigeon airspeed (from Pennycuick et al 2013)
mean.airspeed <- 18.5
sd.airspeed <- 2.28

# square-root transformed step lengths
step.par <- c(0.01,   1.0, 2.5, sqrt(mean.airspeed), # mean of gamma distribution
              0.01,   1.0, 2.5, sqrt(sd.airspeed), # s.d. of gamma distribution
              0.0001, 0.0, 0.0, 0.0) # zero-mass, or zero-inflation

# starting parameters for turning angle
angle.par <- c(pi,  pi,  pi, 0,
               0.2, 0.3, 0.5, 0.8)

# specify distributions for each data stream
dist <- list(step.sqrt='gamma', angle='wrpcauchy')

# initial distribution of states, estimated from step lengths
delta0 <- c(0.75, 0.2, 0.03, 0.02)

# specify formula for state switching probabilities as function of solar time
form <- ~cosinor(solar.time, 24)
```

### Fitting a Hidden Markov model

```
wigeon.hmm <- fitHMM(data=wigeon.df,#[wigeon.df$ID=='5512400-2018-1',], 
                     nbStates=n.states,
                     dist=dist, 
                     Par0=list(step.sqrt=step.par, angle=angle.par), 
                     estAngleMean=list(angle=T),
                     formula=form,
                     delta0=delta0)
```

```
## =======================================================================
```

```
## Fitting HMM with 4 states and 2 data streams
```

```
## -----------------------------------------------------------------------
```

```
##  step.sqrt ~ gamma(mean=~1, sd=~1, zeromass=~1)
```

```
##  angle ~ wrpcauchy(mean=~1, concentration=~1)
```

```
## 
##  Transition probability matrix formula: ~cosinor(solar.time, 24)
```

```
## 
##  Initial distribution formula: ~1
```

```
## =======================================================================
```

```
## DONE
```

```
wigeon.hmm
```

```
## Value of the maximum log-likelihood: -65157.45 
## 
## 
## step.sqrt parameters:
## ---------------------
##              state 1      state 2      state 3    state 4
## mean     0.128664858 1.858785e-01 6.784584e-01 5.94177657
## sd       0.055641704 9.139112e-02 5.206647e-01 3.43782467
## zeromass 0.001096904 8.297645e-05 2.496826e-08 0.00050036
## 
## angle parameters:
## -----------------
##                 state 1   state 2    state 3     state 4
## mean          3.0379532 3.0818308 -3.1311348 -0.01804231
## concentration 0.2437448 0.2235447  0.1759502  0.70656025
## 
## Regression coeffs for the transition probabilities:
## ---------------------------------------------------
##                               1 -> 2     1 -> 3     1 -> 4      2 -> 1
## (Intercept)                -8.205742  15.533307  16.893860 -24.9059645
## cosinorCos(solar.time, 24) -5.504056 -21.802332 -26.561837   1.5919918
## cosinorSin(solar.time, 24) -1.811885   4.432808   6.646195   0.5268574
##                                 2 -> 3    2 -> 4      3 -> 1      3 -> 2
## (Intercept)                -1.68437037 -24.09154 -19.8808075 -0.69363916
## cosinorCos(solar.time, 24)  0.32587907  13.23344   1.8852942  0.35518817
## cosinorSin(solar.time, 24)  0.09330654 -18.32243  -0.2829929 -0.02313476
##                                3 -> 4    4 -> 1    4 -> 2     4 -> 3
## (Intercept)                -3.5941067 -9.697017 -5.696363 -1.3335465
## cosinorCos(solar.time, 24)  1.3532394  5.109482  1.819372 -0.7719263
## cosinorSin(solar.time, 24) -0.3926681 -7.741996  4.757073  1.7081092
## 
## Transition probability matrix (based on mean covariate values):
## ---------------------------------------------------------------
##              state 1      state 2     state 3      state 4
## state 1 1.357191e-19 9.140772e-21 0.002203864 9.977961e-01
## state 2 2.735349e-12 8.818896e-01 0.118110384 5.653650e-17
## state 3 2.597969e-10 2.581013e-01 0.736662320 5.236336e-03
## state 4 2.405604e-07 3.435646e-04 0.362207455 6.374487e-01
## 
## Initial distribution:
## ---------------------
##      state 1      state 2      state 3      state 4 
## 2.615390e-01 7.038665e-06 6.574345e-01 8.101948e-02
```

```
plot(wigeon.hmm, plotTracks=F, sepStates=F, ask=F)
```

```
## Decoding state sequence... DONE
```

```
plotStationary(wigeon.hmm, plotCI=T)
```

```
wigeon.df$state <- viterbi(wigeon.hmm)
ggplot(as.data.frame(wigeon.df), aes(x=ground.speed, y=step, colour=factor(state))) + geom_point() + theme_bw() + scale_colour_viridis_d() + scale_y_sqrt() +
  labs(x='Ground speed [m/s]', y='Step length [km]')
```

```
## Warning: Removed 35 rows containing missing values (geom_point).
```

```
ggplot(as.data.frame(wigeon.df), aes(x=x, y=y)) +
  geom_path(aes(group=ID)) + 
  geom_point(data=wigeon.df[wigeon.df$state!=4,], alpha=0.1, size=0.5, colour='red') +
  theme_bw()
```

How well do the fitted distributions for the four states fit with the actual data?

Plotting it on a map:

## Identifying arrival events

```
# segmentation
wigeon.tracks <- ddply(as.data.frame(wigeon.df), 'ID', function(x){
  migratory <- x$state==4
  x$segment <- c(0, cumsum(diff(migratory)!=0))
  return(x)
})

# alternative:
copy <- wigeon.df
wigeon.df <- wigeon.df[wigeon.df$state!=4,]

wigeon.df <- ddply(as.data.frame(wigeon.df), 'ID', function(x){
  x$dist <- c(0, unlist(lapply(2:nrow(x), function(j){
    distGeo(x[j,c('x', 'y')], x[j-1,c('x', 'y')])/1000
  })))
  
  x$segment <- cumsum(x$dist>threshold.d) + 1
  
  return(x)
})

ggplot(wigeon.df, aes(x=timestamp, y=d2end, colour=segment)) +
  geom_point(size=0.5) + theme_bw() +
  facet_wrap(~ID, scales='free') + 
  scale_colour_viridis_c()
```

```
# retain first arrival at every segment / staging site
arrivals <- ddply(wigeon.df, c('ID', 'segment'), function(x){
  return(x[x$julian==x$julian[1],])
})

arrivals <- arrivals[arrivals$d2start>threshold.d,]

# remove one individual that made within-winter movement (within 52 km from start, February)
arrivals <- arrivals[-which(arrivals$ID=='5512414-2018' & arrivals$segment==1),]
```

```
save(arrivals, file='data/202106_wigeon_arrivals.RData')
load('data/202106_wigeon_arrivals.RData')
```

Brief visual summary of duration of staging events, geodesic distance between arrivals, and overall migration speed:

```
summary.arrival <- ddply(wigeon.df, c('ID', 'segment'), function(x){
  duration <- as.numeric(max(as.Date(x$timestamp)-min(as.Date(x$timestamp))))
  long <- median(x$x); lat <- median(x$y)
  return(data.frame(site=unique(x$site), duration=duration, arrival=min(x$timestamp), last.loc=max(x$timestamp), 
                    julian=min(x$julian), long=long, lat=lat))
})

# add geodesic distance between staging sites
summary.arrival <- ddply(summary.arrival, 'ID', function(x){
  if(nrow(x)>1){
    x$dist.btw <- distGeo(x[,c('long', 'lat')])/1000
    x$time.btw <- c(unlist(lapply(2:nrow(x), function(j){
      as.numeric(difftime(x$arrival[j], x$last.loc[j-1], units='days'))
    })), NA)
    x$speed <- x$dist.btw/x$time.btw
  }else{
    x$time.btw <- x$dist.btw <- NA
    x$speed <- NA
  }
  return(x)
})

summary.speed <- ddply(wigeon.df, 'ID', function(x){
  migratory <- which(wigeon.df$migratory)
  start <- ifelse(min(migratory)>1, min(migratory)-1, 1)
  end <- ifelse(max(migratory)<nrow(x), max(migratory)+1, nrow(x))
  x <- x[start:end,]
  total.dist <- sum(distGeo(x[,c('x', 'y')]), na.rm=T)/1000
  total.dur <- (as.numeric(max(x$timestamp))-as.numeric(min(x$timestamp)))/86400
  
  return(data.frame(site=unique(x$site), dist=total.dist, dur=total.dur, speed=total.dist/total.dur))
})

# all birds:

summary(summary.arrival$duration) # in days
```

```
##    Min. 1st Qu.  Median    Mean 3rd Qu.    Max. 
##   0.000   0.000   1.000   4.784   7.000  58.000
```

```
summary(summary.arrival$dist.btw) # in km
```

```
##    Min. 1st Qu.  Median    Mean 3rd Qu.    Max.    NA's 
##   10.33  120.25  220.86  277.38  376.28 1611.40      35
```

```
summary(summary.speed$speed) # in km/day
```

```
##    Min. 1st Qu.  Median    Mean 3rd Qu.    Max. 
##   2.345  30.871  42.937  53.553  75.863 199.493
```

```
# Dutch birds:

summary(summary.arrival$duration[summary.arrival$site=='Netherlands']) # in days
```

```
##    Min. 1st Qu.  Median    Mean 3rd Qu.    Max. 
##   0.000   0.000   1.000   5.378   8.000  58.000
```

```
summary(summary.arrival$dist.btw[summary.arrival$site=='Netherlands']) # in km
```

```
##    Min. 1st Qu.  Median    Mean 3rd Qu.    Max.    NA's 
##   47.32  152.75  269.50  318.07  426.07 1611.40      20
```

```
summary(summary.speed$speed[summary.speed$site=='Netherlands']) # in km/day
```

```
##    Min. 1st Qu.  Median    Mean 3rd Qu.    Max. 
##   7.431  20.438  38.022  41.547  53.217 117.769
```

```
# Lithuanian birds:

summary(summary.arrival$duration[summary.arrival$site=='Lithuania']) # in days
```

```
##    Min. 1st Qu.  Median    Mean 3rd Qu.    Max. 
##   0.000   0.000   1.000   3.844   6.000  45.000
```

```
summary(summary.arrival$dist.btw[summary.arrival$site=='Lithuania']) # in km
```

```
##    Min. 1st Qu.  Median    Mean 3rd Qu.    Max.    NA's 
##   10.33  101.62  172.14  211.60  253.38  661.18      15
```

```
summary(summary.speed$speed[summary.speed$site=='Lithuania']) # in km/day
```

```
##    Min. 1st Qu.  Median    Mean 3rd Qu.    Max. 
##   2.345  39.761  52.257  69.561  87.358 199.493
```

```
table(summary.arrival$dist.btw>threshold.d)
```

```
## 
## FALSE  TRUE 
##     2   278
```

## Annotation of arrival events with environmental information:

### Convex hull of wigeon tracking data

Below I will derive a convex hull of the tracking data (rather than arrivals), and buffer it by 200 km. I do this to crop and mask the environmental data to the extent of the tracking data.

```
# import tracking data (filtered to only contain stationary periods)
(load('data/202106_migratory_tracks_regular.RData'))
```

```
## [1] "wigeon.tracks"
```

```
proj.tpq <- CRS(paste0("+proj=tpeqd +lat_1=", min(wigeon.tracks$location.lat), 
                       " +lon_1=", min(wigeon.tracks$location.long),
                       " +lat_2=", max(wigeon.tracks$location.lat), 
                       " +lon_2=", max(wigeon.tracks$location.long), 
                " +x_0=0 +y_0=0 +datum=WGS84 +units=m +no_defs +ellps=WGS84 +towgs84=0,0,0"))


po <- SpatialPoints(wigeon.tracks[,c('location.long', 'location.lat')], proj4string=proj.ll)
po.proj <- spTransform(po, CRSobj=proj.tpq)

# derive convex hull encompassing all staging locations and buffer by 200km
hull <- gConvexHull(po.proj)

# buffer the area by 200 km so that  area of interest extends beyond the extent of wigeon locations
# this is necessary to calculate wind conditions for the entire study area
hull.buffered <- gBuffer(hull, width=200000)
hull.ll <- spTransform(hull.buffered, CRSobj=proj.ll)
```

### Import and preparation of environmental data

#### Onset of the thermal growing season

The raster data imported below contain information on the onset of the thermal growing season for the years 1998-2020. These layers were prepared in a separate script, the details of which can be found in Supplementary file 2. Here, I will import the respective layers and mask/crop them using the convex hull for the tracking data. Using the layers from 1998-2017, I will derive the layer TGS\_mean by calculating the average for each raster cell across those years. Using the layers for 2018, 2019, and 2020, I can then derive the TGS\_deviation layers by subtracting the layer containing TGS\_mean from each of the respective layers for TGS\_onset:

```
fil <- list.files('/media/aluco/void/ERA5/t2m_background', pattern='tgs\\.', full.names=T)
years <- paste0('y', gsub('.*tgs\\.', '', gsub('.tif', '', fil)))
tgs.all <- stack(fil); names(tgs.all) <- years

tgs.all <- mask(crop(tgs.all, extent(hull.ll)), hull.ll)

mean.tgs <- calc(subset(tgs.all, 1:20), fun=mean)
sd.tgs <- calc(subset(tgs.all, 1:20), fun=sd)
tgs.dev <- subset(tgs.all, 21:23) - mean.tgs
names(tgs.dev) <- names(tgs.all)[21:23]
```

Let’s plot both TGS\_onset and TGS\_deviation for the study years.

```
## OGR data source with driver: ESRI Shapefile 
## Source: "/media/aluco/void/data/natural_earth/ne_50m_admin_0_countries", layer: "ne_50m_admin_0_countries"
## with 241 features
## It has 94 fields
## Integer64 fields read as strings:  POP_EST NE_ID
```

### Annotation of arrival events with TGS\_onset and TGS\_deviation

```
p.arrival <- SpatialPoints(arrivals[,c('x', 'y')], proj4string=proj.ll)
p.tpq <- spTransform(p.arrival, CRSobj=proj.tpq)

arrivals$x.tpq <- coordinates(p.tpq)[,1]
arrivals$y.tpq <- coordinates(p.tpq)[,2]

arrivals$mean.tgs <- extract(mean.tgs, p.arrival)
arrivals$sd.tgs <- extract(sd.tgs, p.arrival)

arrivals <- ddply(arrivals, 'obs.year', function(x){
  y <- paste0('y', unique(x$obs.year))
  p <- SpatialPoints(x[,c('x', 'y')], proj4string=proj.ll)
  
  x$dev.tgs <- extract(tgs.dev[[y]], p, buffer=500, fun=median)
  x$tgs <- extract(tgs.all[[y]], p, buffer=500, fun=median)
  
  return(x)
})


arrivals <- ddply(arrivals, c('ID', 'segment'), function(x){
  new <- data.frame(date=min(as.Date(x$timestamp)), x=median(x$x), y=median(x$y), x.tpq=median(x$x.tpq),
                    y.tpq=median(x$y.tpq), sex=unique(x$sex), julian=unique(x$julian),
                    year=unique(x$obs.year), capture.site=unique(x$site),
                    mean.tgs=median(x$mean.tgs,na.rm=T), sd.tgs=median(x$sd.tgs, na.rm=T),
                    dev.tgs=median(x$dev.tgs, na.rm=T),
                    tgs=median(x$tgs, na.rm=T), d2start=median(x$d2start))
  return(new)
})

arrivals <- arrivals[!is.na(arrivals$tgs),]

arrivals$delay <- arrivals$julian - arrivals$tgs
arrivals$delay.sc <- scale(arrivals$delay)
```

### Annotation of arrival events GDD accumulated between TGS\_onset and arrival

Growing degree days accumulated since TGS\_onset (>= TGS\_onset):

```
arrivals$obs.year <- factor(gsub('.*-', '', arrivals$ID))

# information about which annotations are available
root <- '/media/aluco/void/ERA5/t2m_background'
fol <- list.files(root, full.names=T)
fol <- fol[!grepl('.tif', fol)]
years <- gsub('/', '', gsub(root, '', fol))

fil <- lapply(fol, function(f){
  list.files(f, pattern='.nc', full.names=T)[1]})
years <- years[sapply(fil, length)>0]

arrivals <- ddply(arrivals, 'obs.year', function(x){
  y <- unique(x$obs.year); #print(as.character(y))
  fil <- rev(list.files(fol[grep(y, fol)], pattern='.nc', full.names=T))
  m <- stack(lapply(fil, stack))
  
  tstamps <- gsub('X', '', names(m))
  tstamps <- unlist(lapply(strsplit(tstamps, '\\.'), function(x){
    paste(paste(x[1], x[2], x[3], sep='-'), paste(x[4], x[5], x[6], sep=':'))
  }))
  tstamps <- as.POSIXct(tstamps, tz='UTC')
  julian <- as.numeric(strftime(tstamps, format='%j'))
  
  m.new <- stack(lapply(sort(unique(julian)), function(d){
    m.tmp <- subset(m, which(julian==d))
    m.tmp <- calc(m.tmp, mean)
    return(m.tmp-273.15)
  }))
  
  names(m.new) <- paste0('d', sprintf('%.3d', sort(unique(julian))))

  p <- SpatialPoints(x[,c('x', 'y')], proj4string=proj.ll)
  x$gdd <- unlist(lapply(1:length(p), function(j){
    get.layers <- paste0('d', sprintf('%.3d', x$tgs[j]:x$julian[j]))
    v <- extract(subset(m.new, get.layers), p[j])
    v <- v-5; v[v<0] <- 0
    gdd <- sum(v)
  }))
  
  x$gdd[x$delay<0] <- -1 * x$gdd[x$delay<0]

  return(x)
})
```

```
## Loading required namespace: ncdf4
```

### Add distance traveled

```
# restrict arrivals to arrivals occurring before July 01
arrivals <- ddply(arrivals, 'ID', function(x){
  july01 <- as.numeric(strftime(paste0(unique(x$year), '-07-01'), format='%j'))
  
  if(any(x$julian<july01)){
    keep <- which(x$julian<july01)
    return(x[keep,])
  }else{
    return(NULL)
  }
})

# add distance traveled (cumulative distance from the first location))
 arrivals <- ddply(arrivals, 'ID', function(x){
   x$mig.cum <- unlist(lapply(1:nrow(x), function(j){
     sum(distGeo(x[1:j,c('x', 'y')])/1000, na.rm=T)
   }))
   return(x)
 })
```

### Scaling of predictors

```
arrivals$individual <- gsub('-.*', '', arrivals$ID)
arrivals$tgs.sc <- scale(arrivals$tgs)
arrivals$x.sc <- scale(arrivals$x.tpq)
arrivals$y.sc <- scale(arrivals$y.tpq)
arrivals$obs.year <- factor(arrivals$year)
arrivals$mig.sc <- scale(arrivals$mig.cum)
arrivals$dev.sc <- scale(arrivals$dev.tgs)
arrivals$gdd.sc <- as.numeric(scale(arrivals$gdd))

arrivals <- ddply(arrivals, c('ID', 'julian'), function(x){
  return(x[nrow(x),])
})
```

### Determine maximum longitude reached by individuals

```
arrivals <- ddply(arrivals, 'ID', function(x){
  x$max.long <- max(x$x.sc)
  x$max.lat <- max(x$y.sc)
  return(x)
})
```

```
save(arrivals, file='data/arrivals_final.RData')
load('data/arrivals_final.RData')
```

### Summary of arrival events

```
## [1] "Number of arrivals:"
```

```
## [1] 208
```

```
## [1] "Number of arrivals per year:"
```

```
## [1] "2018: 111"
```

```
## [1] "2019: 83"
```

```
## [1] "2020: 14"
```

```
## [1] "Number of arrivals per capture site:"
```

```
## [1] "Netherlands: 137"
```

```
## [1] "Lithuania: 71"
```

```
## [1] "Median arrivals per individual and year"
```

```
## [1] 6.5
```

```
## [1] "Median arrivals per individual and year, per study site"
```

```
## [1] "Netherlands: 7"
```

```
## [1] "Lithuania: 4"
```

## Models for wigeon arrival timing at staging sites

### Taking into account repeated observations / longitudinal data

One of the major critical points in our data are repeat observations of individuals during migration; these are of course not independent, and accounting for individual alone might be insufficient. One option would be to only retain the last arrival for each individual. While that would solve the problem, it feels wasteful of the data. Taking into account the correlation between subsequent observations of the same individual should make a for a stronger result, and using both approaches We could use both approaches to strengthen each other (which of course only works if the results correspond).

So, what exactly are is the catch with our data set:

1. We have repeated observations for the same individuals, and for some individuals observations from several year.
2. The repeated observations of individuals might be inherently correlated because individuals have limited speed (i.e. delay at staging site X depends on delay at staging site X-1).

We can first visualise issue No 1) using a simple boxplot:

```
tmp <- arrivals; tmp2 <- arrivals
tmp$individual <- 'all'; tmp$capture.site <- 'all'; 
tmp$divide <- '1'; tmp2$divide <- '2'
#tmp <- rbind(tmp, tmp2)

ggplot(tmp, aes(x=individual, y=delay, fill=capture.site)) + 
  geom_hline(aes(yintercept=0)) +
  geom_boxplot(alpha=1, show.legend=F) + 
  scale_fill_manual(name='', values=c(colour.scheme, all='grey')) +
  scale_y_continuous(name='Delay relative to TGS_onset [days]', breaks=seq(-25,75,25)) +
  theme_bw() + theme(axis.title.x=element_blank()) +
ggplot(tmp2, aes(x=individual, y=delay, fill=capture.site)) + 
  geom_hline(aes(yintercept=0)) +
  geom_boxplot(alpha=0.5) + scale_fill_manual(name='', values=c(colour.scheme, all='grey')) +
  scale_y_continuous(name='Delay relative to TGS_onset [days]', breaks=seq(-25,75,25)) +
  theme_bw() + theme(axis.text=element_blank(), axis.title=element_blank(), 
                     axis.ticks=element_blank(), legend.position='none') + 
  labs(title='Delay relative to TGS_onset') +
ggplot(tmp2, aes(x=capture.site, y=delay, fill=capture.site)) + 
  geom_hline(aes(yintercept=0)) +
  geom_boxplot(alpha=0.5) + scale_fill_manual(name='', values=c(colour.scheme, all='grey')) +
  scale_y_continuous(name='Delay relative to TGS_onset [days]', breaks=seq(-25,75,25)) +
  theme_bw() + theme(axis.text=element_blank(), axis.title=element_blank(), 
                     axis.ticks=element_blank()) + 
  plot_layout(nrow=1, widths=c(0.1, 0.7, 0.2))
```

```
ggplot(tmp, aes(x=individual, y=gdd, fill=capture.site)) + 
  geom_hline(aes(yintercept=0)) +
  geom_boxplot(alpha=1, show.legend=F) + 
  scale_fill_manual(name='', values=c(colour.scheme, all='grey')) +
  scale_y_continuous(name='Growing degree days') +
  theme_bw() + theme(axis.title.x=element_blank()) +
ggplot(tmp2, aes(x=individual, y=gdd, fill=capture.site)) + 
  geom_hline(aes(yintercept=0)) +
  geom_boxplot(alpha=0.5) + scale_fill_manual(name='', values=c(colour.scheme, all='grey')) +
  scale_y_continuous(name='Growing degree days') +
  theme_bw() + theme(axis.text=element_blank(), axis.title=element_blank(), 
                     axis.ticks=element_blank()) + 
  labs(title='Growing degree days at arrival site') +
  plot_layout(nrow=1, widths=c(0.1, 0.9))
```

```
## Warning: Removed 2 rows containing non-finite values (stat_boxplot).

## Warning: Removed 2 rows containing non-finite values (stat_boxplot).
```

This is an issue that is easily accounted for, and we have done it in the previous iterations of the model already by including individual as a random effect in the model. If possible, we should nest observation year in individual to account that individuals had different means in different years

Issue No 2) is a bit trickier to account for, but should be feasible by introducing correlation structures that account for the autocorrelation of repeated observations. To get a better idea of what the problem looks like, let us look at delay over entire spring migrations for individuals with > 1 arrival event, for the year 2018, and the corresponding autocorrelation functions:

We can actually test whether autocorrelation is a problem by setting up a model that does not account for the longitudinal data, simulate residuals from the model, and apply a test specifically designed for that. Let’s try that for the individual with the most arrival events:

```
short.df <- arrivals[arrivals$ID==check.n$ID[which.max(check.n$n)],]

# a simple model with only intercept and individual as random effect
m.corr <- glmmTMB(delay.sc ~ 1, data=short.df)
# simulate scaled residuals from the model
res.sim <- simulateResiduals(m.corr, plot=F, quantreg=T)

# apply test for temporal autocorrelation on recalculated residuals:
testTemporalAutocorrelation(res.sim, time=unique(short.df$julian))
```

```
## 
##  Durbin-Watson test
## 
## data:  simulationOutput$scaledResiduals ~ 1
## DW = 0.38365, p-value = 1.343e-05
## alternative hypothesis: true autocorrelation is not 0
```

Now we can repeat the same model, but will include a correlation structure to account for the autocorrelation in the data. The only problem here is that we cannot actually condition the simulation of residuals on random effects, as this has, at the time of writing, not been implemented in the package *glmmTMB*. As an alternative, I will plot the residuals from both models over observation times:

```
## repeat using a covariance structure

short.df$obs.time <- numFactor(short.df$julian)

# a simple model with only intercept and individual as random effect
m.cov <- glmmTMB(delay.sc ~ ou(obs.time+0|ID), data=short.df)
# simulate scaled residuals from the model
res.cov <- simulateResiduals(m.cov, plot=F, quantreg=T)


new <- data.frame(obs.time=short.df$julian, residuals=c(residuals(m.corr), residuals(m.cov)), 
                  model=c(rep('mod.corr', nrow(short.df)), rep('mod.cov', nrow(short.df))))

ggplot(new, aes(x=obs.time, y=residuals)) +
  geom_path(aes(group=model), size=0.2) + 
  geom_point() + 
  facet_wrap(~model, scales='free_y') + theme_bw()
```

This seems to have worked as intended: while the residuals from the null model seem to show a trend over time, this is not the case for the model with the correlation structure. Including a correlation structure in the models for this project should thus account for auto-correlation within individual and year, or at least reduce the effect of these on the results.

### Models for arrival conditions

Let’s start working on a model that accounts for both issues. As said before, we had already accounted for individuals by including a random effect, so we will extend this by including a correlation structure for longitudinal data. The structure that would come to mind immediately is a autocorrelation structure (AR) for time series - but this assumes that observations are spaced equally in time. This is of course not the case for the arrivals of wigeons. The alternative is an Ornstein-Uhlenbeck correlation structure, which allows for irregular observations, and should (if observations were regular as assumed for the AR structure) be equivalent to the AR structure, or at least approximate it. That should make it a good choice for what we want to achieve.

I will start with a model with only an intercept term and the conditional model composed of a random effect and the correlation structure. Model **a** will have arrival delay as dependent variable, and model **b** the growing degrees accumulated between the TGS\_onset and the arrival of wigeons:

```
# define numerical factor levels for time of observation (this is required for the function to work)
arrivals$obs.time <- numFactor(arrivals$julian)

# base model without fixed effects (just conditional model)
m0a <- glmmTMB(delay.sc ~ 1 + ou(obs.time+0|ID) + (1|obs.year/individual), data=arrivals)
m0b <- glmmTMB(gdd.sc ~ 1 + ou(obs.time+0|ID) + (1|individual), data=arrivals)
```

Arrival delay relative to TGS\_onset

|  | delay.sc | | |
| Predictors | Estimates | CI | Statistic |
| (Intercept) | -0.04 | -0.31 – 0.23 | -0.26 |
| N ID | 32 | | || N individual | 28 | | || N obs.year | 3 | | || Observations | 208 | | |

Growing degree days between TGS\_onset and arrival

|  | gdd.sc | | |
| Predictors | Estimates | CI | Statistic |
| (Intercept) | 0.10 | -0.26 – 0.46 | 0.54 |
| N ID | 32 | | || N individual | 28 | | || Observations | 206 | | |

Now we can investigate whether capture site and sex have any effect on the conditions at staging sites upon arrival:

```
m1a <- glmmTMB(delay.sc ~ capture.site + sex + ou(obs.time+0|ID) + (1|obs.year/individual), data=arrivals)
m1b <- glmmTMB(gdd.sc ~ capture.site + sex + ou(obs.time+0|ID) + (1|obs.year/individual), data=arrivals)
```

Arrival delay relative to TGS\_onset

|  | delay.sc | | |
| Predictors | Estimates | CI | Statistic |
| (Intercept) | -0.31 | -0.94 – 0.32 | -0.98 |
| capture.site [Netherlands] | 0.24 | -0.35 – 0.83 | 0.80 |
| sex [m] | 0.23 | -0.34 – 0.80 | 0.78 |
| N ID | 32 | | || N individual | 28 | | || N obs.year | 3 | | || Observations | 208 | | |

```
##             R2m       R2c
## [1,] 0.01701502 0.9511725
```

Growing degree days between TGS\_onset and arrival

|  | gdd.sc | | |
| Predictors | Estimates | CI | Statistic |
| (Intercept) | -0.42 | -1.24 – 0.40 | -1.01 |
| capture.site [Netherlands] | 0.26 | -0.51 – 1.03 | 0.67 |
| sex [m] | 0.64 | -0.10 – 1.39 | 1.69 |
| N ID | 32 | | || N individual | 28 | | || N obs.year | 3 | | || Observations | 206 | | |

```
##             R2m       R2c
## [1,] 0.05731432 0.9989011
```

Neither sex of the individual nor capture site can contribute much, so I will drop these from the models, while including the terms we are interested in for this study, specifically a) maximum longitude reached, and its interaction with distance traveled. I will further include c) TGS\_deviation (TGS\_onset - TGS\_mean) to account for potential effects of year:

```
m2a <- glmmTMB(delay.sc ~ dev.sc + max.long + max.long:mig.sc:capture.site + capture.site + 0 +
                ou(obs.time+0|ID) + (1|individual/obs.year), data=arrivals)

m2c <- glmmTMB(delay.sc ~ dev.sc + max.long + max.lat:mig.sc + capture.site +
                ou(obs.time+0|ID) + (1|individual/obs.year), data=arrivals)

arrivals.b <- arrivals[!is.na(arrivals$gdd.sc),]
nrow(arrivals.b)
```

```
## [1] 206
```

```
m2b <- try(glmmTMB(gdd.sc ~ dev.sc + max.long + max.long:mig.sc + capture.site +
                ou(obs.time+0|ID) + (1|obs.year/individual), data=arrivals.b))
# this model has trouble converging
ranef(m2b)$cond$obs.year
```

```
##        (Intercept)
## 2018  3.756712e-17
## 2019 -3.684430e-17
## 2020 -7.228238e-19
```

```
# we can see that the estimate for the year of observation is very small
# so, rather than parameterising observation year for each individual separately, I will include it as a fixed effect instead:

m2b <- try(glmmTMB(gdd.sc ~ dev.sc + max.long + max.long:mig.sc + obs.year + capture.site +
                ou(obs.time+0|ID) + (1|individual), data=arrivals.b))
```

Arrival delay relative to TGS\_onset

|  | delay.sc | | |
| Predictors | Estimates | CI | Statistic |
| dev.sc | -0.30 | -0.35 – -0.25 | -12.20 |
| max.long | -0.48 | -0.71 – -0.24 | -3.96 |
| capture.site [Lithuania] | 0.06 | -0.37 – 0.49 | 0.27 |
| capture.site [Netherlands] | 0.52 | 0.17 – 0.87 | 2.91 |
| max.long \* capture.site [Lithuania] \* mig.sc | -0.17 | -0.33 – -0.01 | -2.08 |
| max.long \* capture.site [Netherlands] \* mig.sc | -0.26 | -0.34 – -0.19 | -6.76 |
| N ID | 32 | | || N obs.year | 3 | | || N individual | 28 | | || Observations | 208 | | |

```
##            R2m       R2c
## [1,] 0.4192838 0.9939793
```

Growing degree days between TGS\_onset and arrival

|  | gdd.sc | | |
| Predictors | Estimates | CI | Statistic |
| (Intercept) | 0.51 | -0.16 – 1.19 | 1.50 |
| dev.sc | -0.01 | -0.07 – 0.04 | -0.50 |
| max.long | -0.52 | -0.86 – -0.17 | -2.96 |
| obs.year [2019] | -0.31 | -1.10 – 0.47 | -0.78 |
| obs.year [2020] | -0.17 | -1.70 – 1.36 | -0.22 |
| capture.site [Netherlands] | 0.14 | -0.60 – 0.88 | 0.37 |
| max.long \* mig.sc | -0.27 | -0.36 – -0.18 | -6.19 |
| N ID | 32 | | || N individual | 28 | | || Observations | 206 | | |

```
##            R2m R2c
## [1,] 0.2908686   1
```

What do the effects mean in real terms? We had to scale the model predictors as there were large discrepancies between the scales of the predictors, making it harder to interpret to what extent arrival relative to TGS\_onset changes with maximum longitude and distance traveled. To aid this understanding, we can use the model to predict the outcome for some specific scenarios, and then un-scale the predictions to be able to look at the biological dimension of the effect. Here, we can apply this procedure using hypothetical Dutch wigeons breeding in the Baltic States, around Moscow, Perm, and in the Ob Delta after having migrated over 1,000 km, with an average onset of the thermal growing season (TGS\_deviation = 0):

```
predict.data <- data.frame(
  long=c(capture.sites$long[2], 37.61321, 56.23141, 68.07126), 
  lat=c(capture.sites$lat[2], 55.73124, 57.99333, 66.63664), 
  location=c('Lithuania', 'Moscow', 'Perm', 'Ob delta'),
  distance=1000,
  capture.site='Netherlands', 
  dev.sc=0
)

points.tpq <- spTransform(SpatialPoints(predict.data[,1:2], proj4string=proj.ll), CRSobj=proj.tpq)
predict.data$x <- coordinates(points.tpq)[,1]
predict.data$y <- coordinates(points.tpq)[,2]

# set individual and year to NA to compute population-level estimate:
predict.data$individual <- predict.data$ID <- NA
predict.data$obs.year <- NA

# set observation time to May 01
predict.data$obs.time <- numFactor(121)

# functions to scale distance and maximum longitude using the same parameters as the full data set:
rescale.dist <- function(x){
  (as.numeric(as.character(x)) - attr(sc.mig, 'scaled:center')) / attr(sc.mig, 'scaled:scale')
}

rescale.long <- function(x){
  (as.numeric(as.character(x)) - attr(sc.lon, 'scaled:center')) / attr(sc.lon, 'scaled:scale')
}

# function to un-scale arrival delay:
unscale.delay <- function(x){
  as.numeric(as.character(x)) * attr(sc.delay, 'scaled:scale') + attr(sc.delay, 'scaled:center')
}

predict.data$max.long <- rescale.long(predict.data$x)
predict.data$mig.sc <- rescale.dist(predict.data$distance)

prediction <- predict(m2a, type='response', se.fit=T, re.form=NA, newdata=predict.data)

result <- data.frame(
  breeding.loc=predict.data$location,
  mean.delay.predicted=unscale.delay(prediction$fit),
  standard.error=unscale.delay(prediction$se.fit)
)
print(result)
```

```
##   breeding.loc mean.delay.predicted standard.error
## 1    Lithuania             39.08420       28.92973
## 2       Moscow             33.84031       27.94562
## 3         Perm             26.83373       27.92141
## 4     Ob delta             22.49278       28.68135
```

Let us also take a look at the proportion of deviance explained by the conditional model, and the additional deviance explained by the fixed effects. As the implemented models are gaussian, the function *sigma()* returns the maximum likelihood estimate of the residual standard deviation.

```
# Delay relative to TGS_onset

(dev.null.a <- sigma(glmmTMB(delay.sc ~ 1, data=arrivals)))
```

```
## [1] 0.8058267
```

```
(dev.cond.a <- sigma(m0a))
```

```
## [1] 0.2137367
```

```
(dev.full.a <- sigma(m2a))
```

```
## [1] 0.08747064
```

```
# Residual deviance - proportion explained by conditional model:
1 - (dev.cond.a/dev.null.a)
```

```
## [1] 0.734761
```

```
# Residual deviance - proportion explained by full model:
1 - (dev.full.a/dev.null.a)
```

```
## [1] 0.8914523
```

```
# Growing degree days on arrival

(dev.null.b <- sigma(glmmTMB(gdd.sc ~ 1, data=arrivals)))
```

```
## [1] 0.9915975
```

```
(dev.cond.b <- sigma(m0a))
```

```
## [1] 0.2137367
```

```
(dev.full.b <- sigma(m2a))
```

```
## [1] 0.08747064
```

```
# Residual deviance - proportion explained by conditional model:
1 - (dev.cond.b/dev.null.b)
```

```
## [1] 0.7844522
```

```
# Residual deviance - proportion explained by full model:
1 - (dev.full.b/dev.null.b)
```

```
## [1] 0.9117882
```

### Repeating models with only the last arrival for each individual & year

```
short <- ddply(arrivals, 'ID', function(x){return(x[nrow(x),])})

m3a <- glmmTMB(delay.sc ~ dev.sc + max.long + capture.site + 0 + (1|individual/obs.year), data=short)
m3b <- glmmTMB(delay.sc ~ dev.sc + max.lat + capture.site + 0 + (1|individual/obs.year), data=short)

m3c <- glmmTMB(gdd.sc ~ dev.sc + max.long + capture.site + 0 + (1|individual/obs.year), data=short)
```

|  | delay.sc | | |
| --- | --- | --- | --- |
| Predictors | Estimates | CI | Statistic |
| dev.sc | -0.35 | -0.65 – -0.04 | -2.22 |
| max.long | -0.60 | -0.88 – -0.31 | -4.07 |
| capture.site [Lithuania] | 0.14 | -0.35 – 0.64 | 0.57 |
| capture.site [Netherlands] | 0.68 | 0.24 – 1.12 | 3.02 |
| Random Effects | | | |
| σ2 | 0.52 | | || τ00 obs.year:individual | 0.26 | | || τ00 individual | 0.00 | | || N obs.year | 3 | | || N individual | 28 | | || Observations | 32 | | |

```
##           R2m       R2c
## [1,] 0.444347 0.6278922
```

|  | delay.sc | | |
| --- | --- | --- | --- |
| Predictors | Estimates | CI | Statistic |
| dev.sc | -0.41 | -0.78 – -0.04 | -2.19 |
| max.lat | 0.31 | -0.31 – 0.92 | 0.98 |
| capture.site [Lithuania] | -0.35 | -1.16 – 0.45 | -0.86 |
| capture.site [Netherlands] | 0.05 | -0.90 – 0.99 | 0.10 |
| Random Effects | | | |
| σ2 | 0.59 | | || τ00 obs.year:individual | 0.25 | | || τ00 individual | 0.32 | | || ICC | 0.35 | | || N obs.year | 3 | | || N individual | 28 | | || Observations | 32 | | |

```
##            R2m       R2c
## [1,] 0.1677723 0.5752823
```

|  | gdd.sc | | |
| --- | --- | --- | --- |
| Predictors | Estimates | CI | Statistic |
| dev.sc | -0.25 | -0.72 – 0.22 | -1.06 |
| max.long | -0.79 | -1.22 – -0.36 | -3.62 |
| capture.site [Lithuania] | 0.65 | -0.09 – 1.38 | 1.73 |
| capture.site [Netherlands] | 1.13 | 0.44 – 1.83 | 3.21 |
| Random Effects | | | |
| σ2 | 0.64 | | || τ00 obs.year:individual | 1.05 | | || τ00 individual | 0.00 | | || N obs.year | 3 | | || N individual | 27 | | || Observations | 31 | | |

```
##            R2m       R2c
## [1,] 0.3336815 0.7483033
```
