# Supplementary material for: Migration distance affects how closely Eurasian wigeons follow spring phenology during migration
Source: Mov Ecol. 2021 Dec 11;9:61. doi: 10.1186/s40462-021-00296-0 (PMC8665524; doi:10.1186/s40462-021-00296-0)
Supplement: Supplementary file 3 — Additional file 3. Environmental data preparation (R-code). [file 40462_2021_296_MOESM3_ESM.html]

Additional file 3: Environmental data preparation


# Additional file 3: Environmental data preparation

#### Mariëlle van Toor, Sergey Kharitonov, Saulius Svazas, Jonas Waldenström

#### June 10, 2021

## Description

In this third part, we will prepare the environmental data sets, and annotate arrivals of wigeons at staging sites with the respective environmental information. The data preparation itself is computationally expensive, at least for the processing of climate re-analysis data, and thus beyond the scope of this document. I will however include the code for the steps we have taken to prepare the data so that interested readers can replicate the process should they wish to do so.

With regard to environmental information, we used several the following data sets:

- ECMWF ERA5-Land hourly data, available from 1981 to present, specifically: temperature at 2m above ground, u-wind and v-wind component at 10m above ground (downloaded from Copernicus Climate Data Store)

We used hourly temperature at 2m above ground from the ERA5 climate re-analysis to derive two alternative predictors, specifically the onset of the thermal growing season. Here, we used a reference temperature of 5 C, as literature indicates that this works well to indicate the growth of cold-season plants like grass in temperate and boreal regions.

We calculated the onset of the thermal growing season for 23 years, from 1998 to 2020. We used the years 1998-2017 to produce a long-term average by calculating the mean day marking the onset of the TGS, and the actual study years to reflect the conditions experienced by the wigeons during migration.

## Session set-up

```
# Import relevant packages

# spatial / temporal
library(rgdal) # link to GDAL library
library(lubridate) # time operations
library(geosphere) # spherical trigonometry
library(maptools) # package providing functionality for calculating solar noon
library(rgeos) # link to GEOS library
library(raster) # for working with gridded environmental data

# fitting Hidden Markov Models
library(momentuHMM)

# plotting & misc
library(ggplot2)
library(viridisLite)
library(plyr)
library(patchwork)
library(sf)
library(wesanderson)
library(interactions)

# projection statements used in the analysis
proj.ll <- CRS('+proj=longlat +datum=WGS84')

# wigeon capture sites
capture.sites <- data.frame(site=c('Netherlands', 'Lithuania'), 
                            long=c(4.768945, 21.467045), 
                            lat=c(52.527679, 55.266407), stringsAsFactors=FALSE)

Sys.setlocale("LC_ALL","en_US.UTF-8")
```

```
## Warning in print.momentuHMM.version(): A newer version (1.5.4) is available from
## CRAN
```

```
## Warning in readOGR("/media/aluco/void/data/natural_earth/
## ne_50m_rivers_lake_centerlines/"): Dropping null geometries: 461
```

```
## R version 4.1.2 (2021-11-01)
## Platform: x86_64-pc-linux-gnu (64-bit)
## Running under: Ubuntu 20.04.3 LTS
## 
## Matrix products: default
## BLAS:   /usr/lib/x86_64-linux-gnu/blas/libblas.so.3.9.0
## LAPACK: /usr/lib/x86_64-linux-gnu/lapack/liblapack.so.3.9.0
## 
## locale:
##  [1] LC_CTYPE=en_US.UTF-8       LC_NUMERIC=C              
##  [3] LC_TIME=en_US.UTF-8        LC_COLLATE=en_US.UTF-8    
##  [5] LC_MONETARY=en_US.UTF-8    LC_MESSAGES=en_US.UTF-8   
##  [7] LC_PAPER=sv_SE.UTF-8       LC_NAME=C                 
##  [9] LC_ADDRESS=C               LC_TELEPHONE=C            
## [11] LC_MEASUREMENT=sv_SE.UTF-8 LC_IDENTIFICATION=C       
## 
## attached base packages:
## [1] stats     graphics  grDevices utils     datasets  methods   base     
## 
## other attached packages:
##  [1] interactions_1.1.5 wesanderson_0.3.6  sf_1.0-1           patchwork_1.1.1   
##  [5] plyr_1.8.6         viridisLite_0.4.0  ggplot2_3.3.5      momentuHMM_1.5.3  
##  [9] raster_3.4-13      rgeos_0.5-5        maptools_1.1-1     geosphere_1.5-10  
## [13] lubridate_1.7.10   rgdal_1.5-23       sp_1.4-5          
## 
## loaded via a namespace (and not attached):
##  [1] Brobdingnag_1.2-6   sass_0.4.0          jsonlite_1.7.2     
##  [4] foreach_1.5.1       bslib_0.2.5.1       shiny_1.6.0        
##  [7] assertthat_0.2.1    pander_0.6.4        doRNG_1.8.2        
## [10] yaml_2.2.1          numDeriv_2016.8-1.1 pillar_1.6.1       
## [13] lattice_0.20-45     glue_1.4.2          jtools_2.1.3       
## [16] digest_0.6.27       promises_1.2.0.1    colorspace_2.0-2   
## [19] htmltools_0.5.1.1   httpuv_1.6.1        pkgconfig_2.0.3    
## [22] purrr_0.3.4         xtable_1.8-4        mvtnorm_1.1-2      
## [25] scales_1.1.1        later_1.2.0         CircStats_0.2-6    
## [28] tibble_3.1.2        proxy_0.4-26        generics_0.1.0     
## [31] ellipsis_0.3.2      withr_2.4.2         cli_3.0.1          
## [34] magrittr_2.0.1      crayon_1.4.1        mime_0.11          
## [37] evaluate_0.14       fansi_0.5.0         doParallel_1.0.16  
## [40] MASS_7.3-54         foreign_0.8-81      class_7.3-19       
## [43] tools_4.1.2         lifecycle_1.0.0     stringr_1.4.0      
## [46] munsell_0.5.0       rngtools_1.5        crawl_2.2.1        
## [49] compiler_4.1.2      jquerylib_0.1.4     e1071_1.7-7        
## [52] rlang_0.4.11        classInt_0.4-3      units_0.7-2        
## [55] grid_4.1.2          rstudioapi_0.13     iterators_1.0.13   
## [58] rmarkdown_2.9       boot_1.3-28         gtable_0.3.0       
## [61] codetools_0.2-18    DBI_1.1.1           R6_2.5.0           
## [64] knitr_1.33          dplyr_1.0.7         fastmap_1.1.0      
## [67] utf8_1.2.1          KernSmooth_2.23-20  stringi_1.7.3      
## [70] parallel_4.1.2      Rcpp_1.0.7          vctrs_0.3.8        
## [73] tidyselect_1.1.1    xfun_0.24
```

## Deriving predictors from environmental data

### Define study area using wigeon lcoations

```
# import tracking data (filtered to only contain stationary periods)
(load('data/202106_migratory_tracks_regular.RData'))
```

```
## [1] "wigeon.tracks"
```

```
proj.tpq <- CRS(paste0("+proj=tpeqd +lat_1=", min(wigeon.tracks$location.lat), 
                       " +lon_1=", min(wigeon.tracks$location.long),
                       " +lat_2=", max(wigeon.tracks$location.lat), 
                       " +lon_2=", max(wigeon.tracks$location.long), 
                " +x_0=0 +y_0=0 +datum=WGS84 +units=m +no_defs +ellps=WGS84 +towgs84=0,0,0"))


po <- SpatialPoints(wigeon.tracks[,c('location.long', 'location.lat')], proj4string=proj.ll)
po.proj <- spTransform(po, CRSobj=proj.tpq)

# derive convex hull encompassing all staging locations and buffer by 200km
hull <- gConvexHull(po.proj)

# buffer the area by 200 km so that  area of interest extends beyond the extent of wigeon locations
# this is necessary to calculate wind conditions for the entire study area
hull.buffered <- gBuffer(hull, width=200000)
hull.ll <- spTransform(hull.buffered, CRSobj=proj.ll)
```

### Deriving onset of thermal growing season

Code we used to derive onset of the thermal growing season for the years 1998-2019:

```
# function for estimating tgs
est.tgs.5 <- function(x, d){ # gt is threshold temperature for growing
  cum.t <- cumsum(x-5)
  tgs <- d[which.min(cum.t)]
  return(tgs)
}

# information about which annotations are available
root <- '/media/aluco/void/ERA5/t2m_background'
fol <- list.files(root, full.names=T)
fol <- fol[!grepl('.tif', fol)]
years <- gsub('/', '', gsub(root, '', fol))

fil <- lapply(fol, list.files, pattern='.nc', full.names=T)
years <- years[sapply(fil, length)>0]
fil <- unlist(fil); names(fil) <- years

m <- lapply(years, function(y){
  print(y);
  f.new <- paste0(root,'/tgs.',y,'.tif')
  
  if(!file.exists(f.new)){
    m <- stack(fil[y])
    tstamps <- gsub('X', '', names(m))
    tstamps <- unlist(lapply(strsplit(tstamps, '\\.'), function(x){
      paste(paste(x[1], x[2], x[3], sep='-'), paste(x[4], x[5], x[6], sep=':'))
    }))
    tstamps <- as.POSIXct(tstamps, tz='UTC')
    days <- as.Date(tstamps)
    days <- days[month(days)<8]
    
    m.new <- stack(lapply(unique(days), function(d){
      m.tmp <- subset(m, which(days==d))
      m.tmp <- calc(m.tmp, mean)
      return(m.tmp-273.15)
    }))
    names(m.new) <- strftime(as.character(unique(days)), format='%j')
    
    
    cells <- 1:ncell(m.new)
    rem <- which(is.na(values(m.new[[1]])))
    keep <- which(!is.na(values(m.new[[1]])))
    cells <- cells[-rem]
    
    tgs.values <- unlist(lapply(cells, function(x, m){
      if(x %in% seq(0, ncell(m), 10000)){print(x)}
      p <- SpatialPoints(xyFromCell(m, x), proj4string=CRS(proj4string(m)))
      v <- extract(m, p)
      n <- as.numeric(gsub('X', '', names(m)))
      cum.t <- cumsum(v-5)
      tgs <- n[which.min(cum.t)]
      return(tgs)
    }, m=m.new))
    
    new.values <- rep(NA, ncell(m.new))
    new.values[keep] <- tgs.values
    m.tgs <- m.new[[1]]
    values(m.tgs) <- new.values
    writeRaster(m.tgs, filename=paste0(root,'/tgs.',y,'.tif'))
    rm(list=c('m.new', 'm', 'm.tgs', 'tstamps', 'days'))
    gc()
  }
  return(file.exists(f.new))
})
```
