# Supplementary material for: Migration distance affects how closely Eurasian wigeons follow spring phenology during migration
Source: Mov Ecol. 2021 Dec 11;9:61. doi: 10.1186/s40462-021-00296-0 (PMC8665524; doi:10.1186/s40462-021-00296-0)
Supplement: Supplementary file 4 — Additional file 4. Estimating effect of tagging on arrival timing (R-code). [file 40462_2021_296_MOESM4_ESM.html]

Additional file 4: Estimating effect of tagging on arrival timing


# Additional file 4: Estimating effect of tagging on arrival timing

#### Mariëlle van Toor, Sergey Kharitonov, Saulius Svazas, Jonas Waldenström

#### 7/23/2021

## About this document

This part of the analyses covers the comparison between ring recovery data and the tagged wigeons to estimate potential effects of tagging on their general migratory timing. Ring recovery data were available to us from the schemes Moscow, London, and Arnhem, and we derived the mean and s.d. for recovery dates of ring-marked birds in longitudinal bands with a width of 10 degrees. The analysis was applied to each ringing scheme separately. This part was done using Clipper, and is thus not further detailed here.

Here, we will import both the results from the ring recovery analysis, and the arrival events for tagged wigeons. For each wigeon arrival event, I will calculate the difference between the mean date for ringed birds in a given longitudinal band and the first arrival event that was recorded either in, or beyond that longitudinal band. I will then estimate the delay of tagged wigeons relative to the ringed population, and how delay might be affected by longitude or TGS\_{deviation} using mixed-effects regression.

## Session set-up

```
library(rgdal)

# plotting & misc
library(ggplot2)
library(viridisLite)
library(plyr)
library(interactions)
library(lubridate)

# regression models
library(glmmTMB)
library(sjPlot)
library(interactions)
library(DHARMa)
library(effects)

# projection statements used in the analysis
proj.ll <- CRS('+proj=longlat +datum=WGS84')

# wigeon capture sites
capture.sites <- data.frame(site=c('Netherlands', 'Lithuania'), 
                            long=c(4.768945, 21.467045), 
                            lat=c(52.527679, 55.266407), stringsAsFactors=FALSE)

colour.scheme <- c(Netherlands='#f77d0f', Lithuania='#542788')
```

```
## R version 4.1.2 (2021-11-01)
## Platform: x86_64-pc-linux-gnu (64-bit)
## Running under: Ubuntu 20.04.3 LTS
## 
## Matrix products: default
## BLAS:   /usr/lib/x86_64-linux-gnu/blas/libblas.so.3.9.0
## LAPACK: /usr/lib/x86_64-linux-gnu/lapack/liblapack.so.3.9.0
## 
## locale:
##  [1] LC_CTYPE=en_US.UTF-8       LC_NUMERIC=C              
##  [3] LC_TIME=en_US.UTF-8        LC_COLLATE=en_US.UTF-8    
##  [5] LC_MONETARY=en_US.UTF-8    LC_MESSAGES=en_US.UTF-8   
##  [7] LC_PAPER=sv_SE.UTF-8       LC_NAME=C                 
##  [9] LC_ADDRESS=C               LC_TELEPHONE=C            
## [11] LC_MEASUREMENT=sv_SE.UTF-8 LC_IDENTIFICATION=C       
## 
## attached base packages:
## [1] stats     graphics  grDevices utils     datasets  methods   base     
## 
## other attached packages:
##  [1] effects_4.2-0      carData_3.0-4      DHARMa_0.4.3       sjPlot_2.8.9      
##  [5] glmmTMB_1.1.2      lubridate_1.7.10   interactions_1.1.5 plyr_1.8.6        
##  [9] viridisLite_0.4.0  ggplot2_3.3.5      rgdal_1.5-23       sp_1.4-5          
## 
## loaded via a namespace (and not attached):
##  [1] nlme_3.1-152        insight_0.14.2      numDeriv_2016.8-1.1
##  [4] tools_4.1.2         TMB_1.7.21          backports_1.2.1    
##  [7] bslib_0.2.5.1       utf8_1.2.1          R6_2.5.0           
## [10] sjlabelled_1.1.8    DBI_1.1.1           colorspace_2.0-2   
## [13] nnet_7.3-16         withr_2.4.2         tidyselect_1.1.1   
## [16] emmeans_1.6.2-1     compiler_4.1.2      performance_0.7.2  
## [19] cli_3.0.1           sandwich_3.0-1      bayestestR_0.10.0  
## [22] sass_0.4.0          scales_1.1.1        mvtnorm_1.1-2      
## [25] stringr_1.4.0       digest_0.6.27       minqa_1.2.4        
## [28] rmarkdown_2.9       pkgconfig_2.0.3     htmltools_0.5.1.1  
## [31] lme4_1.1-27.1       rlang_0.4.11        rstudioapi_0.13    
## [34] jquerylib_0.1.4     generics_0.1.0      zoo_1.8-9          
## [37] jsonlite_1.7.2      dplyr_1.0.7         magrittr_2.0.1     
## [40] parameters_0.14.0   Matrix_1.3-4        Rcpp_1.0.7         
## [43] munsell_0.5.0       fansi_0.5.0         lifecycle_1.0.0    
## [46] stringi_1.7.3       multcomp_1.4-17     yaml_2.2.1         
## [49] MASS_7.3-54         grid_4.1.2          sjmisc_2.8.7       
## [52] crayon_1.4.1        lattice_0.20-45     ggeffects_1.1.0    
## [55] splines_4.1.2       jtools_2.1.3        pander_0.6.4       
## [58] sjstats_0.18.1      knitr_1.33          pillar_1.6.1       
## [61] boot_1.3-28         estimability_1.3    effectsize_0.4.5   
## [64] codetools_0.2-18    glue_1.4.2          evaluate_0.14      
## [67] mitools_2.4         modelr_0.1.8        vctrs_0.3.8        
## [70] nloptr_1.2.2.2      foreach_1.5.1       gtable_0.3.0       
## [73] purrr_0.3.4         tidyr_1.1.3         assertthat_0.2.1   
## [76] datawizard_0.1.0    xfun_0.24           xtable_1.8-4       
## [79] broom_0.7.8         survey_4.1-1        coda_0.19-4        
## [82] survival_3.2-13     tibble_3.1.2        iterators_1.0.13   
## [85] TH.data_1.0-10      ellipsis_0.3.2
```

## Ring recovery results

Import results from the analysis of ring recovery data. You will nice that the ring recoveries from the Moscow ringing scheme span a wider range of longitudes than either the London or Arnhem ringing scheme. As we compare ring recoveries with wigeon arrivals falling into the same longitudinal bands, not all of these mean recovery dates will be part of the final comparison.

```
df <- read.delim('../wigeon_consortium/arrival_model/data/sergei/rings_in_spring.csv', header=T, sep=',', as.is=T)
df$mean.julian <- as.numeric(strftime(df$mean.date, format='%j'))
df$ringing.scheme[df$ringing.scheme=='Moskwa'] <- 'Moscow'
df$ringing.scheme[df$ringing.scheme=='Arnhem/Leiden'] <- 'Arnhem'
df <- df[df$species=='wigeon',]

df$long.point[df$ringing.scheme=='London'] <- df$long.point[df$ringing.scheme=='London'] - 1.5
df$long.point[df$ringing.scheme=='Moscow'] <- df$long.point[df$ringing.scheme=='Moscow'] + 1.5
```

## Import arrival events

Import arrival events as detailed in Supplementary file 2, and restrict the data from ring recoveries to the longitudinal extent of observed arrivals.

```
load('data/arrivals_final.RData')
range(arrivals$x)
```

```
## [1]  5.605343 79.537685
```

```
df <- df[df$long.max>min(arrivals$x) &
         df$long.min<max(arrivals$x),]

range(c(df$long.min, df$long.max))
```

```
## [1]  0 80
```

## Merging ring recovery and tracking data

```
new <- ddply(arrivals, 'ID', function(x){
  rbind.fill(lapply(1:nrow(df), function(j){
    y <- df[j,]
    if(any(x$x>y$long.min)){
      jul <- min(x$julian[x$x>y$long.min])
      y$pass <- jul; y$dev.sc <- x$dev.sc[x$julian==jul]
      y$capture.site <- unique(x$capture.site); y$sex <- unique(x$sex)
      y$x.sc <- x$x.sc[x$julian==jul]
      return(y)
    }else{
      return(NULL)
    }
  }))
})
```

## Regression model

Similar to the model described in Supplementary file 2, we here would like to include data in a model that are neither independent nor un-correlated. Consequently, I will again include individual as a random effect, and include a correlation structure to account for the auto-correlation between subsequent observations of the same individual.

```
new$obs.time <- numFactor(new$pass)
new$delay <- new$pass - new$mean.julian
new$individual <- gsub('-.*', '', new$ID)
new$obs.year <- gsub('.*-', '', new$ID)

m01 <- glmmTMB(delay ~ 0 + ringing.scheme + ou(obs.time + 0 | ID) + (1|individual/obs.year), data=new)
tab_model(m01, show.stat=T, show.p=F)
```

|  | delay | | |
| --- | --- | --- | --- |
| Predictors | Estimates | CI | Statistic |
| ringing.scheme [Arnhem] | 8.27 | 2.30 – 14.23 | 2.71 |
| ringing.scheme [London] | 8.11 | 2.14 – 14.08 | 2.66 |
| ringing.scheme [Moscow] | -1.01 | -6.54 – 4.51 | -0.36 |
| N ID | 32 | | || N obs.year | 3 | | || N individual | 28 | | || Observations | 336 | | |

```
# individuals are later than Arnhem & London birds, but ahead of Moscow birds, on average

m02 <- glmmTMB(delay ~ 0 + ringing.scheme + capture.site + sex + x.sc +
                 ou(obs.time + 0 | ID) + (1|individual/obs.year), data=new)
tab_model(m02, show.stat=T, show.p=F)
```

|  | delay | | |
| --- | --- | --- | --- |
| Predictors | Estimates | CI | Statistic |
| ringing.scheme [Arnhem] | 11.53 | -0.53 – 23.59 | 1.87 |
| ringing.scheme [London] | 11.38 | -0.68 – 23.44 | 1.85 |
| ringing.scheme [Moscow] | 3.41 | -8.47 – 15.30 | 0.56 |
| capture.site [Netherlands] | -4.37 | -15.60 – 6.86 | -0.76 |
| sex [m] | 2.91 | -8.09 – 13.90 | 0.52 |
| x.sc | 9.25 | 5.59 – 12.91 | 4.96 |
| N ID | 32 | | || N obs.year | 3 | | || N individual | 28 | | || Observations | 336 | | |

```
# sex and capture site do not seem to contribute much

m03 <- glmmTMB(delay ~ ringing.scheme + ringing.scheme:x.sc + dev.sc +
                 ou(obs.time + 0 | ID) + (1|individual/obs.year), data=new)
tab_model(m03, show.stat=T, show.p=F)
```

|  | delay | | |
| --- | --- | --- | --- |
| Predictors | Estimates | CI | Statistic |
| (Intercept) | 11.61 | 5.79 – 17.42 | 3.91 |
| ringing.scheme [London] | -0.27 | -4.24 – 3.71 | -0.13 |
| ringing.scheme [Moscow] | -6.97 | -10.66 – -3.28 | -3.70 |
| dev.sc | 1.25 | -1.29 – 3.79 | 0.96 |
| ringing.scheme [Arnhem] \* x.sc | 1.21 | -3.68 – 6.10 | 0.49 |
| ringing.scheme [London] \* x.sc | 2.54 | -2.35 – 7.43 | 1.02 |
| ringing.scheme [Moscow] \* x.sc | 14.31 | 10.21 – 18.40 | 6.85 |
| N ID | 32 | | || N obs.year | 3 | | || N individual | 28 | | || Observations | 336 | | |

```
# neither does deviation from the mean oTGS

# final model with ringing scheme in interaction with longitude
m04 <- glmmTMB(delay ~ 0 + ringing.scheme + ringing.scheme:x.sc + 
                 ou(obs.time + 0 | ID) + (1|individual/obs.year), data=new)
tab_model(m04, show.stat=T, show.p=F)
```

|  | delay | | |
| --- | --- | --- | --- |
| Predictors | Estimates | CI | Statistic |
| ringing.scheme [Arnhem] | 11.56 | 5.75 – 17.38 | 3.90 |
| ringing.scheme [London] | 11.30 | 5.48 – 17.12 | 3.81 |
| ringing.scheme [Moscow] | 4.64 | -0.93 – 10.21 | 1.63 |
| ringing.scheme [Arnhem] \* x.sc | 1.19 | -3.76 – 6.13 | 0.47 |
| ringing.scheme [London] \* x.sc | 2.51 | -2.43 – 7.45 | 1.00 |
| ringing.scheme [Moscow] \* x.sc | 14.16 | 10.02 – 18.29 | 6.70 |
| N ID | 32 | | || N obs.year | 3 | | || N individual | 28 | | || Observations | 336 | | |

```
MuMIn:::r.squaredGLMM(m04)
```

```
## Warning: 'r.squaredGLMM' now calculates a revised statistic. See the help page.
```

```
##            R2m       R2c
## [1,] 0.2205302 0.6577597
```

```
plot(simulateResiduals(m04))
```

```
(dev.null <- sigma(glmmTMB(delay ~ 1, data=new)))
```

```
## [1] 22.68822
```

```
dev.cond <- sigma(m01)
dev.full <- sigma(m04)

# Residual deviance - proportion explained by conditional model:
1 - (dev.cond/dev.null)
```

```
## [1] 0.3421922
```

```
# Residual deviance - proportion explained by full model:
1 - (dev.full/dev.null)
```

```
## [1] 0.3991683
```

```
## [1] "wigeon.tracks"
```
